# Soil nutrient management influences diversity, community association and functional structure of rhizosphere bacteriome under vegetable crop production

**DOI:** 10.3389/fmicb.2023.1229873

**Published:** 2023-09-28

**Authors:** Adekunle R. Raimi, Obinna T. Ezeokoli, Rasheed A. Adeleke

**Affiliations:** Unit for Environmental Sciences and Management, North-West University, Potchefstroom, South Africa

**Keywords:** fertilizers, microbial networks, agroecosystem, soil-plant-microbe interactions, 16S rRNA gene sequencing, plant-soil ecology

## Abstract

**Introduction:**

Rhizosphere bacterial communities play a crucial role in promoting plant and soil ecosystem health and productivity. They also have great potential as key indicators of soil health in agroecosystems. Various environmental factors affect soil parameters, which have been demonstrated to influence soil microbial growth and activities. Thus, this study investigated how rhizosphere bacterial community structure and functions are affected by agronomic practices such as organic and conventional fertiliser application and plant species types.

**Methods:**

Rhizosphere soil of vegetable crops cultivated under organic and conventional fertilisers in different farms was analysed using high-throughput sequencing of the 16S rRNA gene and co-occurrence network pattern among bacterial species. The functional structure was analysed with PICRUSt2 pipeline.

**Results:**

Overall, rhizosphere bacterial communities varied in response to fertiliser type, with soil physicochemical parameters, including NH_4,_ PO_4_, pH and moisture content largely driving the variations across the farms. Organic farms had a higher diversity richness and more unique amplicon sequence variants than conventional farms. Bacterial community structure in multivariate space was highly differentiated across the farms and between organic and conventional farms. Co-occurrence network patterns showed community segmentation for both farms, with keystone taxa more prevalent in organic than conventional farms.

**Discussion:**

Module hub composition and identity varied, signifying differences in keystone taxa across the farms and positive correlations between changes in microbial composition and ecosystem functions. The organic farms comprised functionally versatile communities characterised by plant growth-promoting keystone genera, such as *Agromyces*, *Bacillus* and *Nocardioides*. The results revealed that organic fertilisers support high functional diversity and stronger interactions within the rhizosphere bacterial community. This study provided useful information about the overall changes in soil microbial dynamics and how the changes influence ecosystem functioning under different soil nutrient management and agronomic practices.

## Introduction

Soil microbes participate in nutrient cycling, organic matter decomposition and energy flow, which are key in sustaining soil ecosystem functions (Ling et al., 2022). However, soil nutrient management, soil properties and climatic conditions, which vary across geographic locations alter how soil ecosystem function through a possible shift in microbial diversity and functions (Ge et al., 2008; Ahkami et al., 2017; Yang et al., 2021). Substantial responses to changes in land use and soil nutrient build-up have been reported for both plant and soil microbial communities, with potential consequences for ecosystem functioning (Nielsen et al., 2015). Soil microbial diversity is vast and its interactions with other ecosystem components (e.g., vegetation and soil parameters) are complex, which significantly impacts the correlation between soil microbial diversity and ecosystem functions in response to disturbances (Garnica et al., 2020). Thus, insights into soil bacteriome structure and how it is affected by agronomic practices are key to making informed decisions that will promote agroecosystem sustainability.

Currently, organic and conventional fertilizers are the main agro-inputs for improving soil nutrient content (Musyoka et al., 2017). Improved soil nutrient content enhances overall plant health by altering the soil parameters and predisposing the selection of certain rhizosphere bacterial communities with unique ecosystem functions. Consequently, the plant and microbes interactions in the soil, along with the above-ground productivity, are affected by the type of fertilizer applied (Ahkami et al., 2017). Using phospholipid fatty acids, a study has reported that organically managed soil enhanced bacterial and fungal biomass, with an increase in total microbial metabolic activity and soil organic matter. Thus, soil nutrient management types could drastically impact soil ecosystem functioning, through the imbalances caused by fertilizer application and nutrient deposition (Martínez-García et al., 2018).

Furthermore, plant species or genotypes influence the synthesis of unique exudates and extracellular enzymes, which impact microscale spatial patterns in soil bacterial communities and differentially stimulate the growth of specific microbial groups under different ecosystems across geographic locations (Hoch et al., 2019; Yan et al., 2021). Usually, plants exert a selective influence on their rhizo-microbiome to acquire key beneficial traits (Jaramillo et al., 2016), indicating rhizosphere microbial communities are a reflection of plant species and plant-beneficial genetic functions. For example, legumes harbor bacteria with nitrogenases that enhance their N-fixing ability (Lugtenberg, 2015). Similarly, plant-associated microbes, which perform functions such as nitrate reduction, denitrification, nutrient solubilization, and production of phytohormones such as indole acetic acid, ethylene and siderophore are a reflection of the host plant’s needs (Raimi et al., 2017). These microbial functions influence plant productivity and health, which in turn drive soil bacterial diversity and ecosystem functions (Mendes et al., 2013).

Soil parameters vary by geographic location and primarily provide similar conditions that affect the activities of plants and their associated microbiome to soil microbes (Mendes et al., 2013). Studies have shown that factors such as soil pH, carbon, organic matter and nutrient content drive spatial patterns of soil microbial diversity within an ecosystem (Bardgett and Caruso, 2020; Nan et al., 2020). Spatial patterns of soil microbial community due to plant species and fertilizer types and how it relates to bacterial taxonomic and functional profiles remain largely underexplored. Increase in land-use intensification is reported to alter soil microbial composition (Felipe-Lucia et al., 2020) and keystone taxa, which are crucial for microbial community structure and ecosystem functioning (Banerjee et al., 2018). Due to the importance and unique role of keystone taxa, their interactions or loss can trigger a shift in microbiome structure (Yang et al., 2021). Interactions between microbial community members have been widely assessed using microbial co-occurrence network analysis (Li et al., 2018; Xu et al., 2022). Thus, co-occurrence patterns and microbial networks are key to understanding how bacterial community interactions and keystone taxa distribution and functions change under different agronomic practices (Huang et al., 2019; Hernandez D. J. et al., 2021).

Consequently, this study assessed the effects of fertilizer type (organic vs. conventional) and plant species type on the microbial dynamics (including keystone taxa) and ecosystem functions of vegetable rhizosphere soil across different farms. It is hypothesized that fertilizer type in each of the farming systems predisposes the selection of certain rhizobacterial communities, which possess diverse but unique functional gene repertoire of agronomic importance. Results from this study could provide baseline information useful in predictive modeling for manipulating soil bacterial communities to improve agroecosystems. Similarly, the approach used in this study could unravel keystone taxa with unique biomarker potential for monitoring soil health and productivity in a given nutrient management system.

## Experimental procedures

### Description of the study sites and rhizosphere soil sampling

Rhizosphere soils were collected from four vegetable farms located in the North West (farms B and S) and Gauteng (farms J and T) provinces of South Africa. Farm B (26°42′55.0″S 27°04′59.6″E) and farm S (26°47′43.5″S 27°02′18.3″E) are situated in JB marks municipality, while farm J (26°11′40.1″S 28°03′58.3″E) and farm T (26°03′16.0″S 27°40′13.3″E) are in Johannesburg and West Rand District municipalities, respectively. The North West province is characterized as a semi-arid climate while the Gauteng province is classified as a mild climate that is neither humid nor too hot (www.sa-venues.com/weather/). Average annual temperature and rainfall are 22.36°C and 36.6 mm for North West province and 20.64°C and 56.11 mm for Gauteng province. The average annual relative humidity for North West and Gauteng provinces are 37.8 and 50.22%, respectively (Weather and Climate, 2023). Vegetable farming is a major agricultural production in the two provinces, with higher production in the North West compared with the Gauteng province (Macaskill, 2016). The farms have been cultivating various vegetables, including cabbage, lettuce, onions, and spinach for over 2 years on the same plot. The Gauteng province farms (farm J and T) practice conventional farming, using chemical fertilizers, pesticides and fungicides, whereas the farms in the North West province (farm B and S) practice mainly organic farming, utilizing plant and animal waste. The organic farms use compost, green waste, and animal manure (poultry, sheep, goat, and cow), while the conventional farms use chemical fertilizers including NPK fertilizers, urea, ammonium nitrate, diammonium phosphate, and pesticides such as carbamates, organophosphates and pyrethroid (Personal communication).

Sampling was conducted in the summer (October–February) of 2021. Based on visual inspection, rhizosphere soils of healthy vegetables of the same age and size including, *Allium cepa* (onion), *Brassica oleracea* (cabbage), *Lactuca sativa* (lettuce), and *Spinacia oleracea* (spinach), were sampled from each farm as described by Barillot et al. (2013), with some modifications. Briefly, vegetables were uprooted and shaken vigorously to remove bulk soil from the roots. Thereafter, the rhizosphere soil was collected by hand-shaking several undamaged roots to release the adhering soil into a sterile beaker. Five replicate samples were collected for each plant per farm and an equal quantity (50 g each) of the soils from the same vegetable for each farm were pooled to obtain a composite sample. An additional sample of the rhizosphere soil was collected from each farm; a different vegetable type was sampled at each farm, bringing the total number of study samples to 20. Samples were immediately placed in sterile zip-lock bags, transported to the laboratory on ice and stored for further analysis.

### Physicochemical parameters and enzyme analyzes of rhizosphere soil

The soil pH was measured from a 1:2.5 soil-water suspension using a pre-calibrated pH meter (HQ40d Hach, United States), while the electrical conductivity (EC) was determined with an EC meter. The amounts of macronutrients (N, P, K, and Ca), micronutrients (Mn, Fe, Zn, and Cu), heavy metals (Pb, Cd, Cr, Hg, and As) and exchangeable cations (Ca^2+^, Mg^2+^, K^+^, and Na^+^) in the soil were determined using the inductively coupled plasma mass spectrometry (ICP-MS), following standard procedure (Bulska and Wagner, 2016). Other physicochemical properties, such as soil texture (sand, silt, and clay proportion), cation exchange capacity (CEC), moisture and organic matter (OM) content, and the soil enzyme activities, including acid and alkaline phosphatases, β-glucosidase, urease and dehydrogenase activity were analyzed at the Eco-Analytical, North-West University, South Africa, following standardized methods described by the Soil Science Society of South Africa (SSSSA, 1990).

### High-throughput sequencing of 16S rRNA gene

Soil community DNA was extracted using Power Soil DNA extraction kits (Qiagen, Hilden, Germany) following the manufacturer’s instructions. The DNA concentration was measured with a Qubit fluorometer (Invitrogen, Carlsbad, CA, United States) and the integrity was checked using gel electrophoresis with a 1% agarose gel. Extracted DNA was normalized to equimolar concentrations (5 ng/μl) using 0.1 M Tris-HCl (pH 8.5) and the partial 16S rRNA gene (V3-V4 region), a universal barcode for the characterization of bacteria, was amplified in a polymerase chain reaction (PCR) using Illumina-barcoded 341F and 805R primers (Klindworth et al., 2013). The 16S rRNA gene library was prepared as described by van Wyk et al. (2017). Paired-end (2 × 300 bp) sequencing of the gene libraries was performed on the Illumina MiSeq sequencer using the Nextera v3 kit (Illumina Inc., San Diego, CA, United States). Sequencing was performed at the sequencing facility of the Unit for Environmental Sciences and Management, North-West University, Potchefstroom, South Africa.

### Bioinformatics

The 16S rRNA gene sequence reads were demultiplexed and trimmed of barcodes using MiSeq Reporter software (Illumina Inc., San Diego, CA, United States) and then quality checked with FastQC (v. 0.11.5) (Babraham Bioinformatics, UK). Low-quality reads were trimmed with trimmomatic software (v. 0.38) (Bolger et al., 2014) and reads with mean nucleotide base quality score of less than 20 (Phred Q score) were removed. Reads were analyzed using the Quantitative Insight into Microbial Ecology v2 platform (QIIME 2, Release 2020.11; Bolyen et al., 2019). Quality-filtered reads were denoised and clustered into amplicon sequence variants (ASVs) using DADA2 denoiser (Callahan et al., 2016) with a pre-trained classifier of the SILVAngs rRNA gene reference (release 138) (Quast et al., 2013). After singleton removal, the ASV table was rarefied to an even depth before the taxonomic assignment and diversity analyzes in QIIME 2. Alpha diversity indices such as the Shannon-Wiener index, inverse Simpson index, Chao1 richness estimator and phylogenetic diversity were analyzed in R v 4.1.1 (R Development Core Team, 2018).

### Predicted functional metagenomic profile of rhizosphere bacterial communities

The functional metagenomic profile of the absolute abundance of microbial genera was predicted using the Tax4Fun2 package (Wemheuer et al., 2020) in R (R Development Core Team, 2018). Firstly, the ASVs were searched against the 16S rRNA gene reference sequences using the basic local alignment search tool (BLAST) via the *runRefBlast* function. Thereafter, the functional prediction was evaluated using the *makeFunctionalPrediction*, where the ASVs were summarized based on the results of the next neighbor search (Wemheuer et al., 2018). Predicted profiles were annotated based on the Kyoto Encyclopaedia of Genes and Genomes (KEGG) orthology (KO) pathways (Kanehisa et al., 2012). KO terms and metabolic pathways were generated for the ASVs and the relative abundance of the functional genes was calculated. To gain insights into the influence of agronomic practices on bacterial community functional genes, only a few of the KO terms contributing to major ecosystem functions (e.g., organic matter decomposition and biogeocycling), plant growth and nutrient metabolism were investigated.

### Co-occurrence network

Bacterial community co-occurrence patterns were analyzed by constructing ecological networks using the Random matrix theory (RMT)-based method in the molecular ecological network analysis (MENA) pipeline^1^ (Faust and Raes, 2016). To assess the impact of fertilizer type on soil bacterial structure, organic and conventional farm networks were constructed. Network complexity was reduced by using only ASVs present in at least 40% of the samples across the organic and conventional farms and the Pearson correlation coefficient was employed for data transformation. Using a multilevel modularity optimization algorithm, bacterial community groups were identified by clustering networks into modules (Blondel et al., 2008). Node connectivity was established based on among-module connectivity (Pi) and within-module connectivity (Zi); creating four sub-categories of nodes (a) peripheral (Zi < 2.5; Pi < 0.62), (b) connectors (Zi < 2.5; Pi > 0.62), (c) module hubs (Zi > 2.5; Pi < 0.62) and (d) network hubs (Zi > 2.5; Pi > 0.62) (Guimera and Amaral, 2005). The networks were visualized in Gephi v 0.9.2.

### Statistical analyzes

All statistical analyzes were performed in R software (v.4.1.1) unless otherwise stated. The significance for all tests was set at *p* < 0.05 and the data was tested across fixed factors: farms, fertilizer types (organic vs. conventional farms) and vegetable species. Data normality was tested using the Shapiro–Wilk test and non-normal data was transformed with log_10_, square root or sine to fit a normal distribution. Normal or non-normal data were analyzed using parametric or non-parametric tests, respectively. Correlation between soil physicochemical and enzyme activity data was tested using Pearson or Spearman rank correlation for normalized or non-normal datasets. The community structure across the farms, and between organic and conventional farm soil was visualized with a nonmetric multidimensional scaling (NMDS) plot and a cluster dendrogram using vegan and dendextend (v. 1.12.0) (Galili, 2015) packages in R studio. Differences in multivariate space across the fixed factors were performed with Bray-Curtis dissimilarity using permutational multivariate analysis of variance (PERMANOVA) and Permutational test for homogeneity of multivariate dispersion (PERMDISP). Linear Discriminant Analysis (LDA) Effect size (LEfSe) (Segata et al., 2011) was performed to detect differentially abundant genera (Mann–Whitney U test or Kruskal-Wallis test, *p*-value < 0.05; LDA score > 2) between the farms, organic and conventional farms and plant species using microeco package in R software (Liu et al., 2021). Subsequently, the LEfSe results were visualized as bar plots. An indicator species functional analysis was performed for predicted KO terms to detect the most discriminatory KOs between the fixed factors. KO terms with false discovery rate (FDR)-adjusted *p* < 0.05 and an indicator value of > 0.1 were taken to be discriminant for the fixed factors. Redundancy analysis (RDA) was performed in R software to show the physicochemical parameters that best explain the variations in the microbial community composition.

### Data availability statement

The 16S rRNA gene sequence data for this study are available in the sequence read archives (SRA) of the National Centre for Biotechnological Information under a BioProject with SRA accession no PRJNA904574 (https://www.ncbi.nlm.nih.gov/bioproject/904574).

## Results

### Rhizosphere soil physicochemical properties

The soil physicochemical parameters varied across the fixed factors with no particular trend. A near-neutral pH range was observed across the farms, with the conventional farms having a higher pH than the organic farms (Table 1). The moisture and OM content ranged from 1.06–1.42% and 4.98–5.89%, respectively. Organic farms largely had a higher CEC and OM content than conventional farms. Farms mostly had low quantities of microelements and heavy metals, except for Pb and As in farm J (Table 1). Soil texture was mainly classified as sandy-clay-loam for organic farms and sandy-loam for conventional farms. Some of the soil physicochemical properties significantly (Kruskal-Wallis test, *p* < 0.05) differed across the farms (Table 1), while only the differences in OM, clay proportion, CEC, NH_4_, P, and Cu were statistically significant (Mann–Whitney or *t*-test, *p* < 0.05) across the fertilizer types.

**Table 1:** Selected soil physicochemical properties across farms and fertilizer types.

Parameters	Organic		Conventional	
	Farm B	Farm S	Farm J	Farm T
pH (H_2_O)	7.05 ± 0.15^a^	7.52 ± 0.13^a^	7.62 ± 0.6^a^	7.68 ± 1.1^a^
Moisture (%)	1.27 ± 0.2^a^	1.06 ± 0.12^a^	1.42 ± 0.4^a^	1.39 ± 0.2^a^
OM (%)	5.89 ± 0.12^a^	5.37 ± 0.11^b^	4.98 ± 0.19^b^	5.13 ± 0.19^b^
TOC	1.62 ± 0.21^c^	2.44 ± 0.02^b^	2.99 ± 0.48^a^	1.68 ± 0.11^c^
EC (mS cm^−1^)	0.44 ± 0.0^b^	0.44 ± 0.0^b^	0.22 ± 0.0^c^	0.70 ± 0.0^a^
CEC (cmol [+]) kg ^−1^	11.68 ± 0.21^b^	29.29 ± 1.62^a^	8.42 ± 1.12^c^	10.24 ± 1.42^bc^
NO_3_ (mg l^−1^)	135.82 ± 1.72^a^	65.34 ± 1.53^c^	35.96 ± 3.52^d^	125.96 ± 4.39^b^
PO_4_ (mg l^−1^)	10.52 ± 0.44^b^	11.03 ± 1.25^ab^	10.45 ± 1.28^b^	14.19 ± 1.82^a^
NH_4_ (mg l^−1^)	1.04 ± 0.1^b^	1.12 ± 0.09^b^	1.53 ± 0.1^b^	4.28 ± 0.44^a^
SO_4_ (mg l^−1^)	35.54 ± 1.32^c^	70.13 ± 2.17^a^	15.37 ± 2.17^d^	53.79 ± 3.03^b^
HCO_3_ (mg l^−1^)	15.25 ± 0.48^b^	42.71 ± 1.41^a^	36.61 ± 3.13^a^	36.61 ± 3.94 ^a^
P (mg kg ^−1^)	42.5 ± 3.27^b^	14.3 ± 1.73^c^	72.2 ± 3.25^a^	40.2 ± 2.34^b^
K (mg l^−1^)	17.2 ± 0.97^bc^	19.55 ± 0.94^ab^	16.42 ± 1.81^c^	53.56 ± 1.81^a^
Na (mg l^−1^)	16.78 ± 0.68^b^	16.78 ± 0.87^b^	10.28 ± 1.56^c^	30.81 ± 2.53^a^
Ca (mg l^−1^)	32.86 ± 2.61^b^	33.26 ± 1.89^b^	13.63 ± 3.56^c^	51.7 ± 3.61^a^
Mg (mg l^−1^)	18.21 ± 1.02^a^	17.26 ± 0.89^a^	6.2 ± 1.24^b^	17.5 ± 2.20^a^
Cl (mg l^−1^)	42.19 ± 2.1^b^	30.14 ± 3.09^c^	12.76 ± 2.6^d^	99.62 ± 3.76^a^
Fe (mg l^−1^)	0.51 ± 0.02^a^	0.0 ± 0.00^c^	0.01 ± 0.0^bc^	0.03 ± 0.0^b^
Cu (mg l^−1^)	0.03 ± 0.00^c^	0.05 ± 0.00^b^	0.19 ± 0.00^a^	0.05 ± 0.00^b^
Zn (mg l^−1^)	0.0 ± 0.00^c^	0.0 ± 0.00^c^	0.14 ± 0.00^a^	0.01 ± 0.00^b^
Pb (mg/kg)	5.46 ± 0.18^d^	53.29 ± 0.91^b^	91.27 ± 0.24^a^	8.25 ± 0.10^c^
As (mg/kg)	2.68 ± 0.16^c^	3.69 ± 0.0b	12.35 ± 0.61^a^	3.47 ± 0.0^b^
Hg (mg/kg)	0.04 ± 0.0^b^	0.11 ± 0.0^b^	2.91 ± 0.18^a^	0.05 ± 0.0^b^
Pd (mg/kg)	0.26 ± 0.02^b^	0.37 ± 0.0^a^	0.17 ± 0.0^c^	0.18 ± 0.0^c^
Cd (mg/kg)	0.03 ± 0.0^c^	0.085 ± 0.0^b^	0.16 ± 0.0^a^	0.02 ± 0.0^c^
Particle size (>2 mm)	3.8 ± 0.78^c^	29.9 ± 2.43^a^	5.2 ± 0.68^b^	0.4 ± 0.00^d^
Clay (%)	23.1 ± 0.75^a^	27.4 ± 2.56^a^	12.6 ± 0.86^c^	17.9 ± 1.76^b^
Sand (%)	65.4 ± 2.72^b^	64.0 ± 3.91^b^	76.3 ± 2.66^a^	65.8 ± 4.3^b^
Silt (%)	11.5 ± 0.86^b^	8.6 ± 1.84^a^	11.1 ± 1.72^b^	16.3 ± 1.8^c^

Values are mean ± SD (*n* = 4). EC, electrical conductivity; CEC, cation exchange capacity; OM, Organic matter; TOC, Total organic carbon. Values with different superscript letters for each farm (across rows) are significantly different based on the non-parametric Kruskal-Wallis H test or the parametric analysis of variance (ANOVA) test.

### Soil enzyme activities

The soil enzyme activities were significantly (Kruskal-Wallis H test, *p* < 0.05) different across the farms and fertilizer type, except for alkaline phosphatase, which was not influenced by the fertilizer type. Mostly, the enzyme activities were higher for organic farms compared to conventional farms (Table 2). The alkaline and acid phosphatases had a very low range of activities across the farms. Comparing among farms, farm B had the highest dehydrogenase and β-glucosidase activities, while farm S had the highest activity for urease. Farm J had the lowest urease activity, which is approximately five, 12 and 23 folds lower compared to farms B, T, and S, respectively (Table 2). The main effect of crop type and the interactions between the fixed factors on soil enzyme activities were not statistically significant (Kruskal-Wallis H test, *p* > 0.05).

**Table 2:** Important rhizosphere soil enzyme activities across the farms.

Parameters	Organic		Conventional	
	Farm B	Farm S	Farm J	Farm T
Dehydrogenase	249.4 ± 7.09^a^	156.28 ± 6.61^c^	171.17 ± 2.1^b^	89.39 ± 0.78^d^
β-glucosidase	140.98 ± 2.41^a^	124.53 ± 0.47^b^	108.09 ± 1.19^c^	83.28 ± 0.22^d^
Alkaline phosphatase	0.26 ± 0.01^b^	0.52 ± 0.04^a^	0.52 ± 0.02^a^	0.26 ± 0.02^b^
Acid phosphatase	0.51 ± 0.02^a^	0.52 ± 0.02^a^	0.52 ± 0.00^a^	0.26 ± 0.01^b^
Urease	10.83 ± 0.48^c^	49.81 ± 1.74^a^	2.17 ± 0.06 ^d^	25.99 ± 0.24^b^

Values are mean ± SD (*n* = 4) followed by superscript letters across the rows. The superscripts are significant differences based on the non-parametric Kruskal-Wallis H test or parametric independent sample ANOVA test across the farms. The unit of measurement for the enzyme are INF μg g^−1^ 2 h^−1^ (dehydrogenase), P-nitrophenol μg g^−1^ h^−1^ (β-glucosidase, acid and alkaline phosphatase) and NH_4_-N μg g^−1^ 2 h^−1^ (urease).

### Diversity and community structure of bacterial amplicon sequence variants

**Figure 1:**
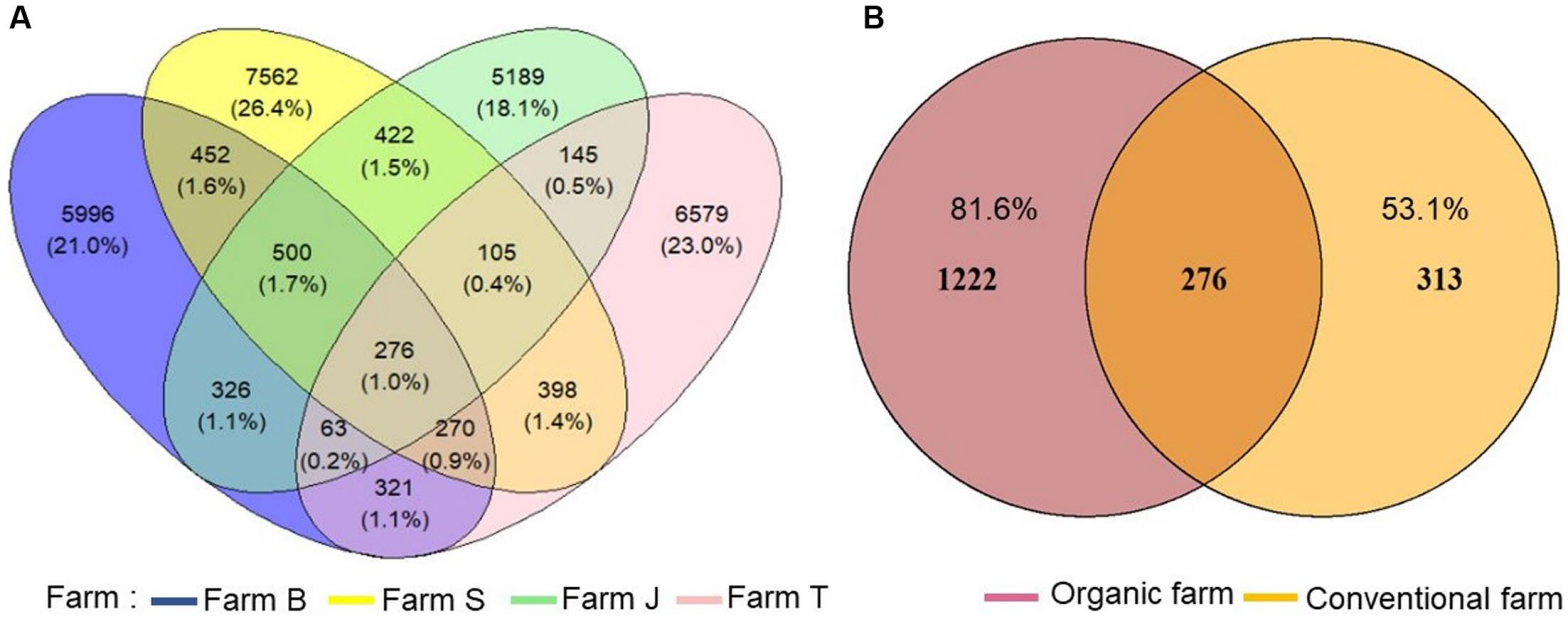
Comparison of unique and shared amplicon sequence variants (ASVs) richness. **(A)** ASV richness across the farms, **(B)** unique ASVs between organic and conventional farms.

A total of 1,933,349 16S rRNA gene reads was obtained from all samples, with a mean read count of 96,667 per sample. Quality-filtered reads were clustered into 28,604 ASVs after pruning low count and low variance features. The ASVs were rarefied to an equal depth of 74,170 (rarefaction curve in Supplementary Figure S1) before examining the treatment effects. The distribution and richness of ASVs differed substantially across the farms, with farm S having the highest number of unique ASVs (Figure 1A). Observed ASVs were higher in organic compared to conventional farms and 276 ASVs were shared among all the farms (Figures 1A,B). Comparing the shared ASVs in the organic (Supplementary Figure S2A) with the conventional (Supplementary Figure S2B) farms, 1,222 and 313 ASVs were exclusive to the organic and conventional farms, respectively (Figure 1B). Similarly, ASV richness differed across the rhizosphere of each vegetable species. Unique ASVs were higher in cabbage, followed by lettuce and onion rhizosphere, while spinach and lettuce rhizosphere share more ASVs (Supplementary Figure S3).

The alpha diversity indices measured were not significantly influenced by the main effect of fertilizer type and plant species, except for the inverse Simpson index, which was significant (ANOVA, *F* = 3.65, *p* = 0.035) across the farms, with the pairwise comparison (Tukey HSD, P-adjusted = 0.0218) showing differences between farms T and S. In addition, the combined effects of the fixed factors (fertilizer type and plant species) and their interactions had no significant effect (general linear model, *p* > 0.05) on the alpha diversity indices measured.

**Figure 2:**
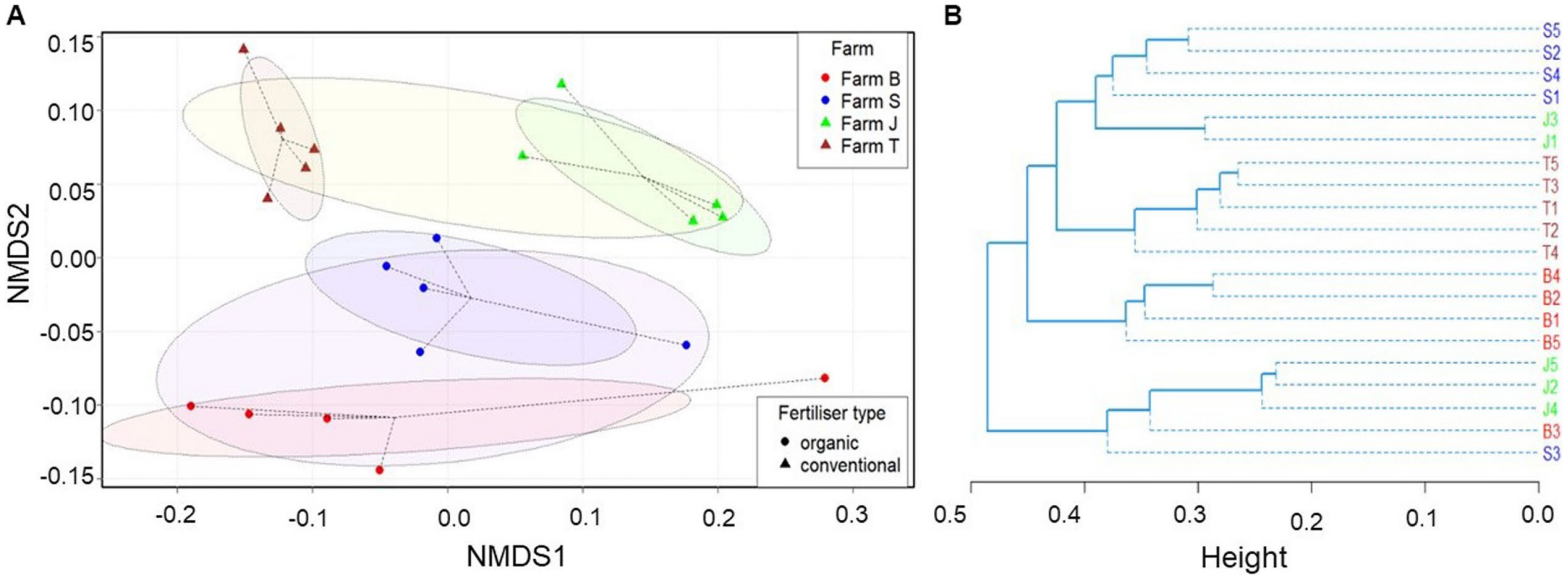
Bray-Curtis dissimilarity between bacterial communities. **(A)** Comparison of observed ASVs between organic and conventional fertilization and across farms based on non-metric multidimensional scaling (NMDS). **(B)** Unweighted paired group mean arithmetic (UPGMA) hierarchical cluster dendrogram. The eclipses in the NMDS plot show 95% confidence intervals (standard error) in multivariate space within the group centroids, while the dotted lines indicate the distance of each sample to its group centroid in multivariate space. The stress plot (Supplementary Figure S5B) for the NMDS showed that the original dissimilarities are well preserved (stress = 0.165047) in the reduced number of dimensions.

Bacterial community structure was highly differentiated across the farms and between the organic and conventional farms in multivariate space. Although the bacterial community structure in the organic farms (farms B and S) are somewhat differentiated, they are jointly less similar to conventional farms (farms J and T) (Figure 2A); the community structural pattern is also supported by the hierarchical clustering (Figure 2B). Moreover, the bacterial community composition was significantly different between the farms (PERMANOVA, *p* = 0.001) and across organic and conventional farms (PERMANOVA, *p* = 0.009), contributing about 39.01 and 11.74% of the variations in the models and a PERMDISP of 0.073 and 0.269, respectively. Significant variation was observed between farms B and T, J and T, and S and T. However, the plant species had no significant (PERMAOVA, *p* = 0.568) effect on the bacterial community structure, which was not clearly differentiated (Supplementary Figure S4). The non-significant and low dispersion rate in the bacterial community across the farms (Supplementary Figure S5A) suggests the results depicted by the NMDS plot in Figure 2A are reliable. The stress plot run for the NMDS plot is below 0.2 (Supplementary Figure S5B). The effect of fertilizer type and plant species interaction on the bacterial community structure was not significant (Supplementary Table S1).

### Dominant and differentially abundant rhizosphere bacterial communities

**Figure 3:**
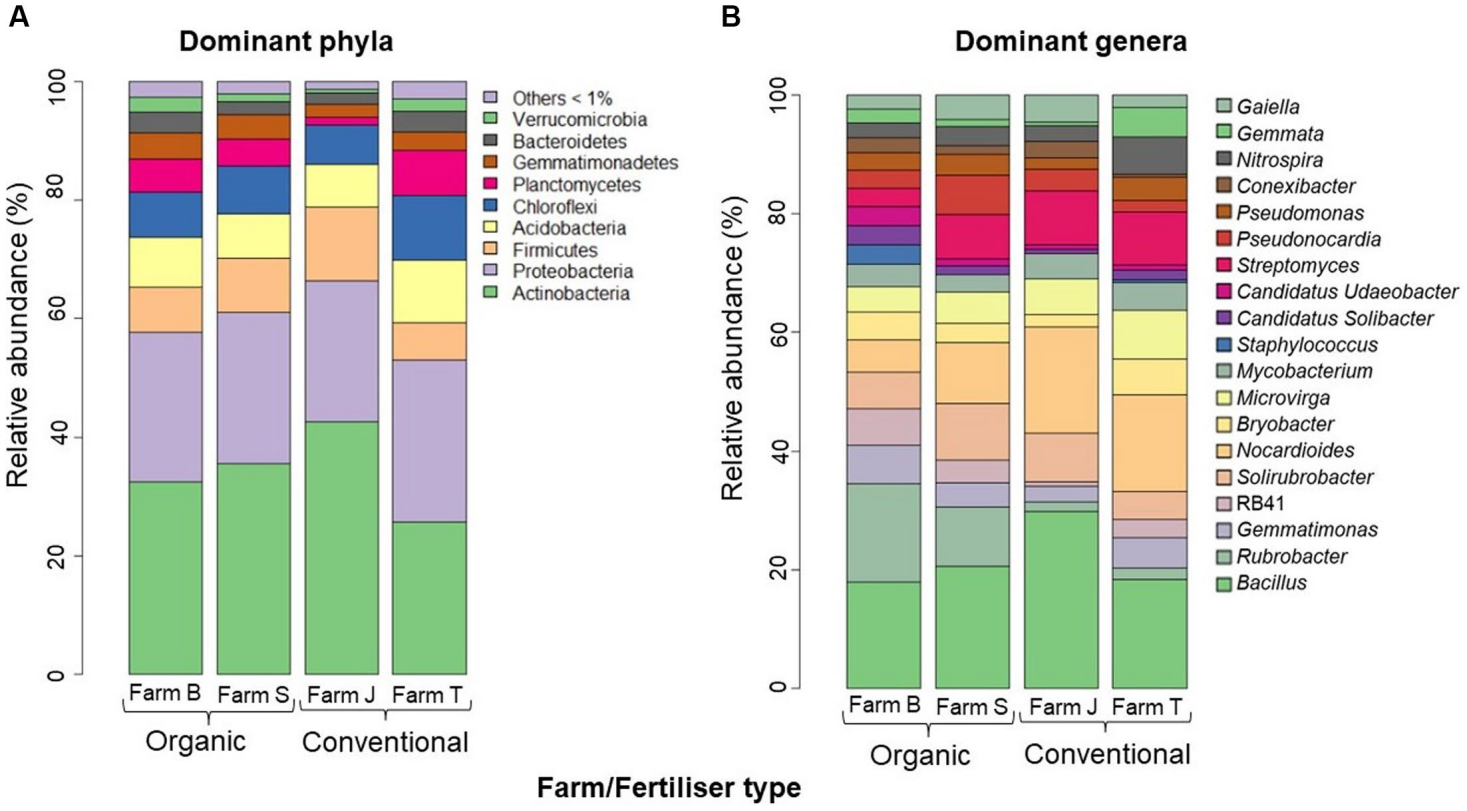
Average relative abundance (>5%) of dominant phylotypes across the farms. **(A)** Dominant Phyla **(B)** Dominant Genus taxa. The phylotypes with average relative abundance below 1% and the unculturable and unclassified at the genus taxa level were expunged from the plot. The bar plots were constructed based on the average relative abundance per farm site in R studio.

Across all datasets, the relatively more abundant classifiable ASVs belonged to nine phyla and 19 major genera. Across the farms, the most relatively abundant (>5%) phyla were Actinobacteria (32.3%), Proteobacteria (25.4%), Acidobacteria (8.4%), Firmicutes (7.6%), Chloroflexi (7.6%), and Planctomyces (5.7%) (Figure 3A). The relatively more abundant (> 5%) genera across the farms are *Bacillus*, *Rubrobacter*, *Gemmatimonas*, *Solirubrobacter*, RB41, *Nocardioides* and *Bryobacter* (Figure 3B). *Bacillus* and *Nocardioides* were relatively more abundant in conventional farms, with their highest abundance found in farm J, while *Rubrobacter* and *Solirubrobacter* were more abundant in organic farms, mainly in farms B and S, respectively. Across the plant species, the bacterial community composition at the phylum and genus taxa levels were not particularly different; however, at the phylum level, Chloroflexi, Planctomycetes and Gemmatimonadetes were relatively most abundant in cabbage, whereas Actinobacteria, Proteobacteria and Firmicutes were the most abundant phyla in onion (Supplementary Figure S6A) compared to other vegetables. Cabbage had the highest relative abundance of *Bacillus* and *Rubrobacter*, whereas onion had the highest relative abundance of *Nocardioides*, *Streptomyces* and *Solirubrobacter* (Supplementary Figure S6B).

**Figure 4:**
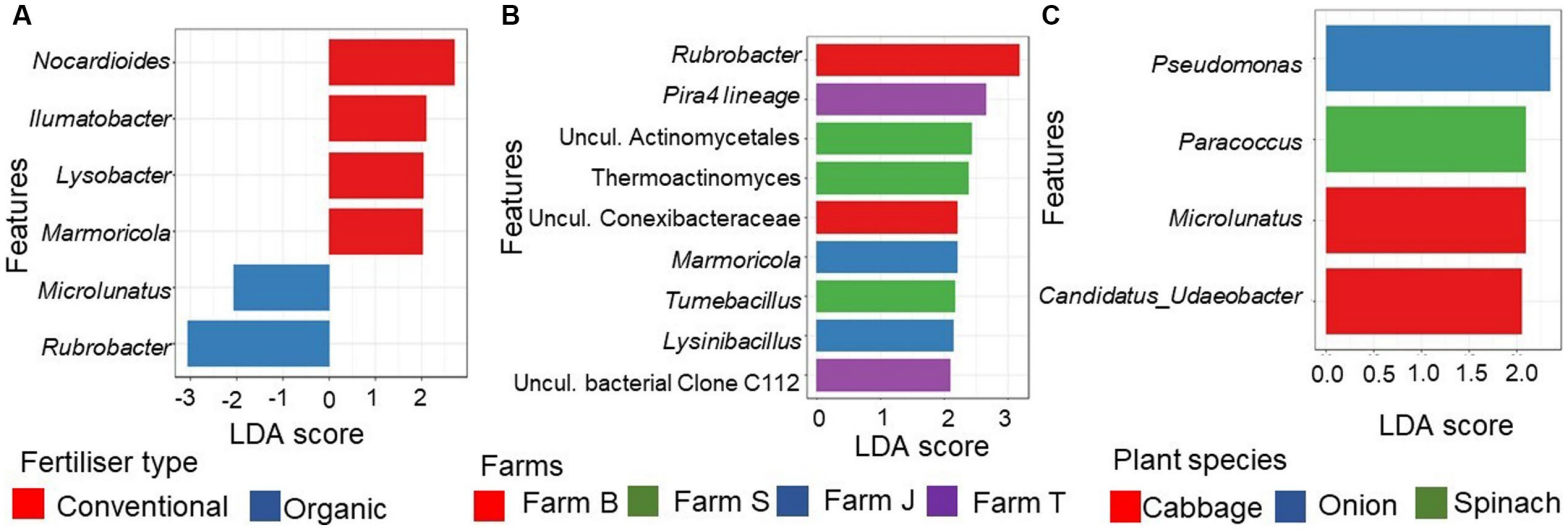
Differentially abundant genera of the rhizosphere bacterial communities. **(A)** Top 6 statistically significant discriminant (FDR-adjusted *p*-value < 0.1, LDA score > 2.4) genera between organic and conventional farms **(B)** Top 9 discriminant (FDR-adjusted *p*-value < 0.05, LDA score > 2.0) between the farms. **(C)** Differentially abundant genera (FDR-adjusted *p*-value < 0.1, LDA score > 2.0) between the plant species. The bar plot was generated using the Linear Discriminant Analysis (LDA) Effect size (LefSe) in the R using the microeco package. Uncul., uncultured.

Linear discriminant analysis (LDA) effect size (LEFSe) showed that a total of 205 features were discriminant across the farms (Kruskal-Wallis H rank-sum test, *p* < 0.05; LDA score > 2.0; Supplementary Table S2) while only 18 features were discriminant for organic farms vs. conventional farms (LDA score > 2.4; Supplementary Table S3). No taxa was discriminant across the plant species after multiplicity adjustment (Kruskal-Wallis rank-sum test; *p* > 0.05). The genera *Rubrobacter* and *Microlunatus* were highly discriminant in organic farms (Figure 4A), while *Nocardioides*, *Ilumatobacter*, *Lysobacter*, and *Marmoricola*, were significantly discriminant in conventional farms (Figure 4A). Some of the bacterial communities that were significant and differentially abundant (FDR-adjusted *p* < 0.05, LDA score > 2.0) at the genus taxa across the farms are shown in Figure 4B. *Rubrobacter* and uncultured Conexibacteraceae were more discriminant in farm B, while Pira4lineage and uncultured bacterial Clone C112 were discriminant in farm T. In farm S, Uncultured Actinomycetales, Thermoactinomyces, and *Tumebacillus* were found to be discriminant. The discriminant features in conventional farms, *Marmoricola* and *Lysobacter,* were mainly from farm J. Before multiplicity adjustment of the LEFSe results for plant species type, *Pseudomonas*, *Paracoccus* and *Candidatus Udaeobacter* were discriminant (*p* > 0.05, LDA score > 2.0) and abundant in cabbage, onion, and spinach, respectively (Figure 4C), while lettuce rhizosphere soil showed no discriminant feature.

### Bacterial functional profile and differentially abundant agroecological important enzymes

**Figure 5:**
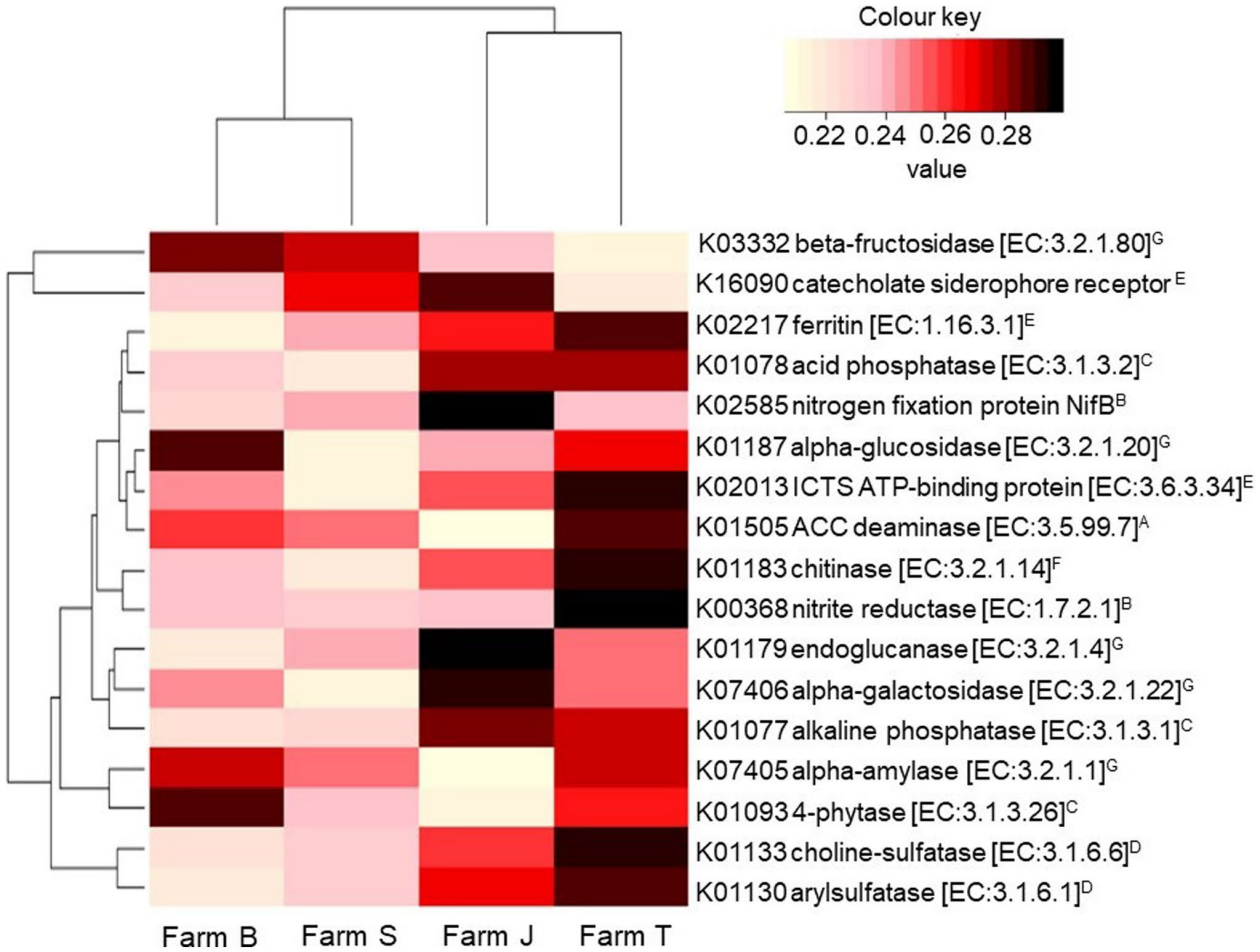
Relative abundance of KEGG Orthology terms contributing to important agroecosystem functions. The hierarchical clustering dendrograms are based on the mean Bray-Curtis distance between the farms. The color range for the relative abundance is scaled across the farms. The superscripts by the enzyme commission (EC) number indicate (^A^); ACC, (^B^); N-fixation, (^C^); phosphorous mineralization, (^D^); sulfur mineralization (^E^); soil iron balance, (^F^); biocontrol, and (^G^); soil C breakdown. ICT, iron complex transport system.

The KO terms and pathways predicted for all the ASVs were 6,429 and 279, respectively. After multiplicity adjustment, 17 of the predicted pathways were significant for fertilizer type factor (Kruskal-Wallis rank sum test, *p* < 0.05; LDA > 0.181; Supplementary Table S4), while no significant pathway was observed for the plant species factor. Across the farms, a total of 161 functions were significant (Kruskal-Wallis rank sum test, *p* < 0.05; LDA > 0.274) after multiplicity adjustment (Supplementary Table S5). Seventeen abundant KO terms, contributing to important agroecological processes such as the synthesis or metabolism of N, P, C, S, and Fe compounds were compared across the farms (Figure 5). Predicted KO terms, including nitrogenase, acid and alkaline phosphatase, ferric chelate reductase and aminocyclopropane-1-carboxylate (ACC) deaminase, had low relative abundance in organic compared to conventional farms. Based on the Bray-Curtis distance, the predicted pathways in organic farms and conventional farms clustered separately as shown by the hierarchical cluster dendrogram plot in Figure 5.

A higher relative abundance of K01505, K02217, K07405, K01187, and K03332 was predicted in the rhizosphere of cabbage relative to other plant species. Similarly, K00368, K02585, K01183, K07406, and K02013 were higher for ASVs from spinach than in other plant species, while onion had the least abundance of predicted KOs (Supplementary Table S6). The major functional profile group of the bacterial communities at taxa rank level 1 is metabolism, followed by environmental information, and genetic and cellular processes. Using LEfSe, it was revealed there are differences in the abundances of key enriched pathways across the fixed factors (Kruskal-Wallis rank-sum test, *p* < 0.05, LDA *>* 0.2). Organic farms had more differentially abundant pathways compared to conventional farms. Amino acid, arginine, proline metabolism, C-fixation and citrate cycle pathways were discriminant in the organic farm (Supplementary Table S4), while sulfur, phosphonate and phosphinate metabolism were more abundant in the conventional farms. Farm T, followed by farm J had the highest number of important enriched pathways (Supplementary Table S5). Xenobiotic degradation and terpenoid and polyketide metabolism were differentially abundant in farm J while bacterial secretion, translation and genetic information system were more abundant in farm T (Supplementary Table S5).

### Relationship between biological and physicochemical properties

Although there were no significant correlations between some of the soil enzyme activities and physicochemical parameters (Supplementary Table S7), beta-glucosidase had a significantly positive correlation with pH (*r* = 0.32, *p* = 0.001) and PO_4_ (*r* = 0.12, *p* = 0.045) but negative correlation with OM (*r* = −0.69, *p* = 0.015), NH_4_^+^ (*r* = −0.72, *p* = 0.005) and K (*r* = −0.30, *p* = 0.019). Both alkaline and acid phosphatase significantly (*p* < 0.05) correlated with TOC, positively and Cl, negatively. Moreover, alkaline phosphatase correlated positively with NO_3_ while acid phosphatase correlated positively with pH and EC. In addition, dehydrogenase had significant correlations that were negative with pH, PO_4_, NH_4_ and K but positive with OM and TOC. Although there exists either a positive or negative correlation between the soil enzymes and moisture content, Ca and sand, the correlations were not significant. Similarly, though not significant, there were correlations between the species function percentage of bacterial community with the soil parameters (Supplementary Figure S7).

**Figure 6:**
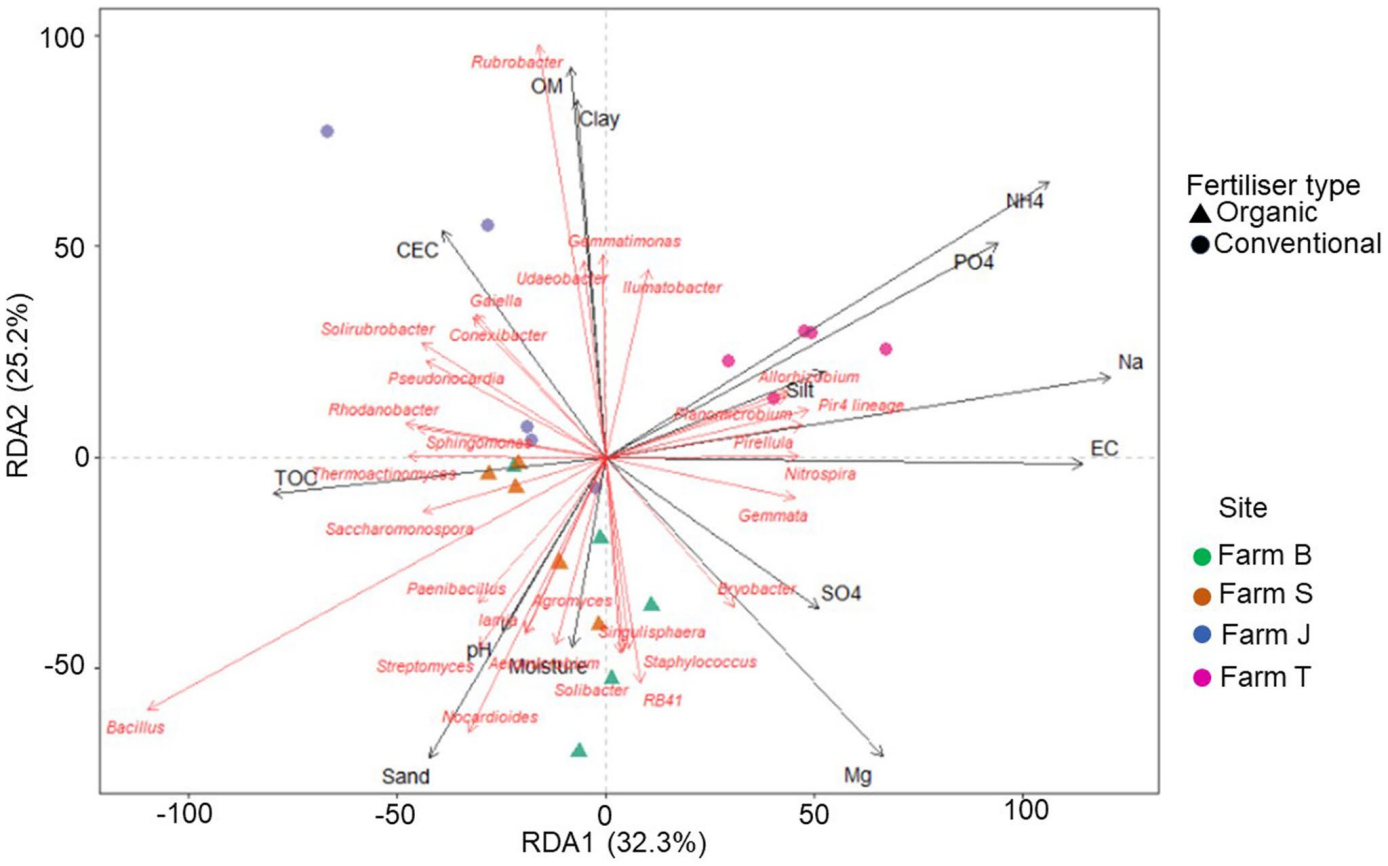
Redundancy analysis (RDA) triplot showing the correlation between bacterial community relative abundance (for 30 major genera) and explanatory variables (physicochemical factors). The first species axis (RDA) is significant (*p* < 0.05).

The RDA plot showed that the soil physicochemical properties contributed 34.5% (R-squared adjusted value) of the variation in the bacterial community structure. Farms and fertilizer type-specific clustering were observed, especially for the organic farms (Figure 6). Among the physicochemical parameters in the RDA model, TOC, OM, EC, and CEC were statistically significant (ANOVA, *p* < 0.05) (Supplementary Table S8). Similarly, there were significant correlations between the environmental variables and the distance matrix (Mantel, *p* < 0.05) (Supplementary Table S8). Overall, the plot showed that soil physicochemical properties largely influenced the variations observed in the bacterial community (Figure 6). PO_4_, Na, and NH_4_ had more impact on the bacterial community structure in farm T, while moisture, TOC, sand, and pH, had more influence on bacterial community structure in the organic farms (farms B and S).

### Co-occurrence analysis

**Figure 7:**
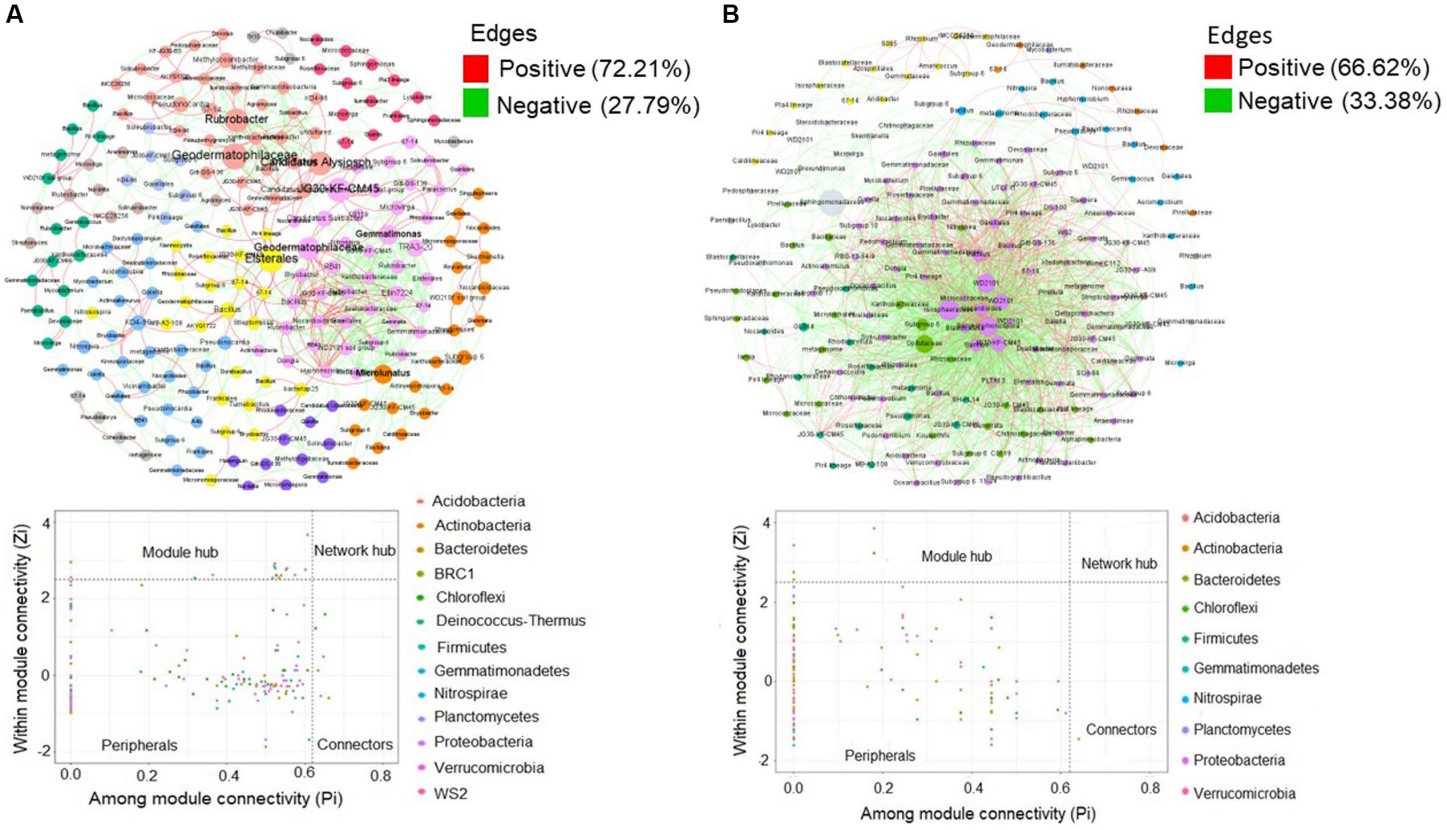
Co-occurrence networks of bacterial communities and classification of nodes for detection of keystone taxa within ecological networks in **(A)** organic and **(B)** conventional farm rhizosphere soil. The nodes represent bacterial species (round shape), colored according to the community modularity class. Node sizes are proportional to their degree of distribution, and nodes having less than two connections have been removed. Edges are network connections signifying significant (FDR-adjusted *p* < 0.01) associations between nodes. Positive associations are colored red, while negative associations are colored blue. Detected module hubs in the two farm networks are linked with Acidobacteria, Actinobacteria, Proteobacteria and Chloroflexi, while the connectors are affiliated with Actinobacteria for both organic and conventional farms. The co-occurrence network statistics are presented in Table 3.

Some of the ASVs had significant (*r* = 0.70, *p* < 0.05) positive correlations with the environmental traits, which produced a significant (Mantel, *p* = 0.001) effect on the network connectivity. The significant ASVs and node connectivity were found to be significant for two major phyla: Actinobacteria (Mantel, *r* = 0.120, *p* = 0.022) and Acidobacteria (Mantel, *r* = 0.344, *p* = 0.009). From the topological metrics (Table 3), both organic and conventional farm networks were non-random as inferred from the significant power-law distribution for each network (*R*^2^_organic_ = 0.88, *p* < 0.05; *R*^2^_conventional_ = 0.84, *p* < 0.05) and the higher values of some of the structural features in the empirical compared to the random networks (Table 3). The empirical network consisted of 247 nodes and 704 edges (Figure 7A) for the organic farms and 284 nodes and 1,027 edges for the conventional farms (Figure 7B).

**Table 3:** Topological properties of empirical and random networks between bacterial communities of organic and conventional farm.

Network indices	Organic		Conventional	
	ENI	100 RNI	ENI	100 RNI
Average clustering coefficient	0.129	0.043 ± 0.006	0.216	0.309 ± 0.011
Average path distance	6.183	3.467 ± 0.038	2.791	2.545 ± 0.027
Geodesic efficiency	0.215	0.321 ± 0.003	0.456	0.435 ± 0.003
Harmonic geodesic distance	4.648	3.115 ± 0.025	2.191	2.300 ± 0.017
Centralization of degree	0.073	0.073 ± 0.000	0.326	0.326 ± 0.000
Centralization of betweenness	0.122	0.074 ± 0.008	0.067	0.090 ± 0.010
Centralization of stress centrality	17.228	0.299 ± 0.028	2.725	0.590 ± 0.055
Centralization of eigenvector centrality	0.210	0.184 ± 0.015	0.175	0.173 ± 0.003
Density	0.019	0.019 ± 0.000	0.050	0.050 ± 0.000
Reciprocity	1.000	1.000 ± 0.000	1.000	1.000 ± 0.000
Transitivity	0.192	0.061 ± 0.006	0.131	0.254 ± 0.005
Connectedness	0.801	0.958 ± 0.021	0.471	0.980 ± 0.017
Efficiency	0.981	0.984 ± 0.000	0.900	0.952 ± 0.001
Hierarchy	0.000	0.000 ± 0.000	0.000	0.000 ± 0.000
Lubeness	1.000	1.000 ± 0.000	1.000	1.000 ± 0.000
Modularity	0.676	0.406 ± 0.006	0.210	0.166 ± 0.004

ENI, Empirical Network Indices; RNI, Random Network indices.

A higher level of positive (~72%) associations occurred among the bacterial communities in the organic farms compared to conventional farms, which had a higher negative (~33%) association. Though community segmentation was apparent in both networks, the organic farms had a more profound fragmentation. Moreover, a higher network complexity was observed for organic farms compared to conventional farms, which had more numbers of smaller modules and edges than organic farms. The module hubs were not of similar parameters, with organic farms (Figure 7A) having more bacterial communities compared to conventional farms (Figure 7B). Actinobacteria, Acidobacteria, Proteobacteria and Chloroflexi were dominant in the keystone nodes. No network hub was observed for both networks. While Actinobacteria occurred as a major node that highly connects modules in both networks (Figure 7), Bacteroides, Proteobacteria, Acidobateria and Chloroflexi are other connectors in the organic farm network. The keystone nodes comprised the genera *Bacillus*, *Nocardioides*, *Blastocatella*, and *Saccharomonsospora*. A major connector in conventional farms is *Agromyces* while organic farms are dominated by the family Micrococcaceae, Roseiflexaceae, Blastocatellaceae, Xanthobacteraceae, and Chitinophagaceae (Figure 7).

## Discussion

Soil microbes significantly contribute to ecosystem functions through organic matter decomposition, nutrient cycling, and mineralization. These functions and plant ecosystems are influenced by agronomic practices such as soil nutrient management and cropping systems. Thus, this study provided insights into the rhizosphere soil bacterial community structure and functions of vegetable crops cultivated under organically and conventionally managed soil in different farms. Functional profiling and differentially abundant taxa of rhizosphere bacteria allowed for the identification of rare microbial communities with unique potentials in agroecosystems, emphasizing the benefits of understanding soil microbiome dynamics to reveal specific patterns, functions and strategies used by microbes under different agronomic conditions.

### Soil physicochemical parameters influence nutrient richness

The fact that soil nutrients and structure drive plant growth and soil microbial diversity is well established (Bach et al., 2010; Xu et al., 2022). Some of the soil parameters, including pH, EC, moisture content, NH_4_^+^ and NO_3_^−^ were not significant across the farms, However, except for pH and P, the measured values were relatively not within the optimal range required for the productivity of most crops, including vegetables (Rosen and Eliason, 2005; Warncke et al., 2009; Liu and Hanlon, 2018). Contrary to our results, low moisture content has been reported in chemically fertilized soils (Rachwał et al., 2021) and a higher pH in organically managed soil (Diacono and Montemurro, 2010; Aziz et al., 2017). Higher levels of organic matter increase soil water holding capacity through soil aggregation, signifying soil organic matter and moisture content are strongly correlated (Rajkai et al., 2015). Although organic farms typically have higher organic matter content and soil texture type that support high moisture content, our results showed that conventional farms had higher moisture content, possibly due to other factors such as time of sampling and irrigation system. Compared to conventional farms, soils in the organic farms largely had higher clay content, CEC and OM content; this is in agreement with the observations from other studies (Han et al., 2016; Rigane et al., 2020). Among the farms, farm S, which is organically managed, had the highest particle size (Table 1). Soil particle sizes impact soil aeration and structure, while high clay content and CEC strongly drive the fixation of key soil nutrients (Ramos et al., 2018; Kome et al., 2019). Being negatively charged, clay particles adsorb positive ions such as Ca^2+^, Fe^3+^, K^+^, and NH_4_^+^ which are key indicators of soil nutrient richness (Tomašić et al., 2021). In contrast, our results show that in the organic farms (Farm B and S), which had higher clay content, some of the positive ions, including NH_4_^+^, K^+^, and Ca^2+^ were relatively lower suggesting other factors, including irrigation, could have influenced the soil nutrient levels.

### Soil enzyme activities in organically and conventionally managed soil

High activity of soil enzymes, including dehydrogenase, β-glucosidase and urease activities have been reported in organic farms (Hernandez T. et al., 2021; Pittarello et al., 2021). Extracellular enzyme production has been positively correlated with high microbial biomass and diversity in the community. Such reports are consistent with our results that showed a high activity of β-glucosidase across organic farms, validating the comparatively higher C mineralization in organic farms compared to conventional farms; compost and manure applied in organic farms are rich in carbon substrate (Zang et al., 2018; Cordero et al., 2019). Acid and alkaline phosphatase activities are optimal at pH 3.0–5.5 and 8.5–11.5, suggesting the low activities across the farms may be linked to the soil neutral pH (Neina, 2019). Soil enzyme activities dynamically respond to soil nutrient management, which influences soil parameters, particularly pH, OM, moisture content and nutrient content (Pan et al., 2013; Koishi et al., 2020). A significant correlation between soil pH and dehydrogenase, β-glucosidase and acid phosphatase suggests pH is a key factor driving enzyme activities (Pan et al., 2013). The observed positive correlation of β-glucosidase with OM indicates the specificity of the enzyme to soil C, which is a fraction of the total soil OM (Meena and Rao, 2021). Corroborating with our result of reduced enzyme activities in the conventional farm, excessive use of chemical fertilizers has been reported to hinder soil enzyme activities (Pittarello et al., 2021; Rachwał et al., 2021). Thus, the types of fertilizers used in plant cultivation affect changes in soil conditions, such as enzyme activities which may cause variations in the dynamics of the associated soil microbial community (Liu and Hanlon, 2018; Ullah et al., 2019).

### Rhizosphere bacterial community diversity and structure

Soil properties, especially organic matter drive bacterial growth and metabolism, suggesting the bacterial richness across the farms is related to soil conditions (Peltoniemi et al., 2021; Rachwał et al., 2021). This was evidenced in the RDA triplot (Figure 6), which revealed a significant correlation between the soil parameters and some bacterial communities. Soil physical and biological parameters, including OM, moisture and nutrient content, pH, dehydrogenase, β-glucosidase, and urease, support improved soil and plant health, which in turn influence the rhizosphere bacteria composition (Yan et al., 2021). Rhizosphere microbiome has some analogous traits with its associated plants, suggesting plant selective impact on rhizosphere microbes and consequently shaping rhizobacterial assemblage (Ling et al., 2022). Similar to other studies, though only the inverse Simpson diversity index was significant, the differences in alpha diversity across the farms suggest fertilizer types influence bacterial community richness (Hu et al., 2011; Acharya et al., 2021). According to Hermans et al. (2017) and Kamaa et al. (2011), bacterial communities respond to prevailing soil nutrient management types, which vary across landscapes; this is in agreement with our results which revealed differences in bacterial communities across the different farms, as depicted by the NMDS plot (Figure 2A). However, the bacterial community structure of soils from similar fertilizer types showed some differences, an indication that other site-specific factors, such as the source and type of seeds, fertilizers, and irrigation systems not considered in this study, may be responsible.

Important bacterial phyla, including Actinobacteria, Proteobacteria, Acidobacteria, Firmicutes and Chloroflexi, which were relatively abundant across the farms have been reported in similar studies (Ezeokoli et al., 2020; Zhang et al., 2022). These phyla usually exist in C-rich niches, such as the rhizosphere, which supports high metabolic activities and fast microbial growth. The variation in taxa abundance across crop species may be due to differences in root architecture and exudates, which differentially stimulate the growth of rhizosphere microbial communities (Hoch et al., 2019; Yan et al., 2021). Similarly, this may have influenced the higher ASVs abundance observed in cabbage compared to other crop species, suggesting the need to further explore factors influencing rhizobacteria diversity variability in different crop species under similar conditions. Several studies have identified core bacterial microbiomes primarily using taxonomic assignment; however, for detailed knowledge of microbial dynamics, it has become imperative to further identify soil microbes using key functions that are commonly expressed (Ling et al., 2022). For instance, Gemmatimonadetes, a recently classified phylum found among the major phyla in the cabbage soil have species that contribute to C-fixation through chlorophototrophy (Thiel et al., 2018). In addition, predominant genera, such as *Bacillus*, *Nocardioides*, *Pseudomonas*, *Rubrobacter*, and *Lysobacter,* which are constantly enhanced in the rhizosphere, contribute to key microbial functions and processes, such as biogeochemical cycling, organic matter decomposition, mineralization and processes that are crucial to agroecosystem sustainability (Raimi et al., 2017; Adeleke et al., 2019).

### Ecological functions of differentially abundant rhizosphere bacterial community

The differentially abundant *Rubrobacter* in organic farms is underexplored despite its ecological restoration and engineering potential, which is attributed to its ability to survive in low-nutrient soil and under intense desiccation (Baubin et al., 2021). Similarly, *Lysin*i*bacillus* and *Pseudomonas* predominant in organic farm soils are well-known plant growth-promoting rhizobacteria involved in N-fixation, phytohormone production and P solubilization (Li et al., 2017; Vignesh et al., 2021; Shahwar et al., 2023). *Candidatus Udaeobacter*, a differentially abundant genus in the spinach rhizosphere, exhibits multidrug resistance and the ability to evade the harmful effects of antimicrobials (Willms et al., 2020). The unique properties of these genera suggest they are suitable indicator species for assessing the soil nutrient status. In contrast to the organic farms, conventional farms had differentially abundant *Norcardioides*, *Lysobacter*, *Ilumatobacter*, and *Marmoricola*. *Nocardioides* participate in bioremediation, especially organochlorine degradation such as lindane pesticide and chloroaromatics, making them a crucial candidate for soil remediation strategy (Ito et al., 2019; Singh and Singh, 2019). *Lysobacter* is a well-known biocontrol agent (Lin et al., 2021) while *Ilumatobacter* and *Marmoricola* participate in C and N cycling, respectively (Edgmont et al., 2012; Liu et al., 2023). Although soil microbes have been extensively classified, the high proportion of unclassified sequences in this study gave credence to the fact that the majority of soil microbiomes are yet to be fully identified and characterized (Delgado-Baquerizo, 2019; Furtak et al., 2020). However, metagenomics, a leading and relatively recent technique for effective analysis of diverse environments has advanced microbial diversity knowledge, offering valuable strategies for optimizing the cultivation of yet uncultured species (Kulski, 2016; Mendes et al., 2017).

Nitrogenase and NifT play key roles in the N-cycle, while cellulases break down cellulose and polysaccharide, and chitinase degrades chitins, contributing to C and N levels in the ecosystem (Glick, 2012). Corroborating the report by Ling et al. (2022), alpha-glucosidase and beta-fructokinase were highly predicted in organic farms, possibly due to the high level of C supplied by compost and animal manure, suggesting a more sustainable soil microbial community may be achieved through organic amendments that increased these key enzymes. Bacterial communities such as *Bacillus*, *Enterobacter*, *Citrobacter*, and *Pseudomonas* detected have been previously reported with N-fixing ability due to their nitrogenases (Gomez-Garzon et al., 2017; Li et al., 2017). On the contrary, nitrite reductase genes driving the denitrification pathway in the N-cycle were significantly abundant in Farm T, which is a conventional farm, establishing the fact that these genes play a crucial role in preventing groundwater pollution caused by nitrate compounds due to excessive chemical fertilizer application (Liu et al., 2020). Arylsulfatase and choline-sulfatase drive the sulfur content in the soil and are usually activated under sulfur-deficient conditions (Cregut et al., 2013; Sánchez-Romero and Olguin, 2015). Surprisingly, these genes were highly prevalent in conventional farms (Farm T and J) (Figure 5), suggesting other factors, including available sulfur, forms of sulfur compounds and other nutrient complexes in the soil may have influenced the prevalence of these enzymes (Siwik-Ziomek et al., 2016). In addition, the high abundance of enzyme coding genes predicted in the rhizosphere of cabbage compared to other vegetables could enhance soil nutrient richness and in turn drive increased soil microbial composition and diversity (de Bruijn, 2015; Xu et al., 2022). While variations across geographic locations drive changes in soil physicochemical parameters (Moore et al., 2022), the changes may in turn influence soil microbial and enzyme activities (Meena and Rao, 2021), suggesting the reason for some of the variations observed across the farms. Thus, it may be recommended toward best practices to always understand agronomic soil parameters for appropriate selection of efficient soil nutrient management.

### Microbial co-occurrence network and keystone taxa

Microbial co-occurrence structure greatly impacts community assembly, abundance and diversity (Pan et al., 2021). Soil nutrient management (types of fertilizer applied) and plant species have been reported to influence the network structure of soil bacterial communities (Xue et al., 2018; Xu et al., 2022). The network analysis showed that conventionally managed soil had high numbers of bacterial communities with negative links, which may indicate weak connections among microbial associations. This observation was corroborated by Huang et al. (2019), who attributed the distinctly weakened ecological interactions between soil microbes to long-term chemical fertilization. In contrast, the higher level of positive interactions in the organic farms may signify greater ecological cooperation among the microbial communities for diverse ecosystem functions and consequently; a network structure with high stability and high-order level complexity (Xue et al., 2018; Gu et al., 2019). Profound community fragmentation was noticed for organic farm networks. According to Hernandez D. J. et al. (2021), ecological stress greatly influences the complexity and stability of microbial networks, indicating fragmentation may not necessarily be due to fertilizer types. Thus, other factors such as soil types, irrigation and land preparation that impact soil microbial diversity may be influential.

In microbial networks, modules comprise species, which are interconnected with more frequent and intensive interactions than other parts of the community (Ling et al., 2022). Compared to conventional farms, the organic farms had high numbers of complex topological structures and modules, which suggests high niche differentiation in organic farms (Figure 7; Table 3). Unique microbes play key roles in the functioning of bacterial communities by contributing to the information current across the entire network (Banerjee et al., 2018). Similar to our observation, Gu et al. (2019) reported no network hubs in their study, suggesting unique phylotypes for specific functions are absent. However, some generalists, including *Bacillus*, *Nocardioides, Agromyces*, and *Blastocatella* were detected, signifying diverse keystone species that could drive the soil microbial communities in each of the farms (Gu et al., 2019). Module hub, network hub and connectors harbor keystone communities whose identification further improves the understanding of microbial community structure and interactions (Pan et al., 2021). In this study, *Bacillus* found in organic farms is a unique generalist, participating in nutrient solubilization and mineralization; therefore, can generate a niche for other plant growth-promoting rhizobacteria. On the other hand, the conventional farm network has *Agromyces* as a major connector, which is involved in xylan degradation; thus, may create a niche for microbial populations that cannot degrade polysaccharides (Rivas et al., 2004).

To further improve our agroecosystem management knowledge, we recommend that the impact of soil nutrient management on soil microbial diversity and functional structure be investigated across seasons. Data on crops cultivated in the previous seasons, irrigation systems and sources of fertilizers were not available, contributing to some of the limitations in this study.

## Conclusion

This study revealed the soil bacterial community diversity and functions are influenced by farm sites and fertilizer types. Unique bacterial communities, which contribute to ecosystem functions were also differentiated across these fixed factors. To a large extent, the discovery of unique bacterial communities such as *Bacillus* and *Rubrobacter* with plant growth-promoting and niche-creation potentials across the farms could improve the understanding of soil microbial dynamics for enhancing soil and plant productivity. In addition, conventional farms had reduced keystone taxa compared to organic farms, signifying fertilizer types impact the abundance of keystone taxa, which is a key factor in ecosystem functioning. This study provided comprehensive details on rhizosphere soil bacterial diversity and functions, which could be useful in the identification of biomarker species for monitoring soil productivity in a particular nutrient management system. Keystone taxa, such as *Bacillus* and *Agromyces* could be a good source of microbial resources for bioformulation production. Overall, the findings provide a baseline for understanding how fertilizer types, organic and conventional fertilizers, impact soil bacteria community structure and related ecological functions. Such knowledge may be useful for monitoring invasive species or formulating a strategy for replenishing extinct beneficial species following land-use intensification or a particular agronomic practice.

## Data availability statement

The datasets presented in this study can be found in online repositories. The names of the repository/repositories and accession number(s) can be found below: https://www.ncbi.nlm.nih.gov/, PRJNA904574.

## Author contributions

RA: conceptualization, methodology, validation, formal analysis, investigation, resources, writing: review and editing, project administration. AR: validation, resources, writing—original draft, review and editing, methodology, validation, formal data analysis, and investigation. OE: validation, data analysis, writing—review and editing. All authors contributed to the article and approved the submitted version.

## Funding

This research was funded by the National Research Foundation (NRF) South Africa, under grant number 116251 and the Technology Innovation Agency with grant number NWU SF2210012/TIA1396/01.

## Conflict of interest

The authors declare that the research was conducted in the absence of any commercial or financial relationships that could be construed as a potential conflict of interest.

## Publisher’s note

All claims expressed in this article are solely those of the authors and do not necessarily represent those of their affiliated organizations, or those of the publisher, the editors and the reviewers. Any product that may be evaluated in this article, or claim that may be made by its manufacturer, is not guaranteed or endorsed by the publisher.

## Footnotes

^1^ http://ieg4.rccc.ou.edu/mena
