# Supplementary material for: Soil nutrient management influences diversity, community association and functional structure of rhizosphere bacteriome under vegetable crop production
Source: Front Microbiol. 2023 Sep 28;14:1229873. doi: 10.3389/fmicb.2023.1229873 (PMC10568080; doi:10.3389/fmicb.2023.1229873)
Supplement: Supplementary file 1 [file Data_Sheet_1.docx]

***Supplementary Materials***

**Title: Soil nutrient management drives diversity, community association and functional structure of rhizosphere bacteriome under vegetable crop production**

**Authors**: Adekunle Raimi, Obinna T. Ezeokoli, Rasheed Adeleke ^*^

**Affiliation**

Unit for Environmental Sciences and Management, North-West University, Potchefstroom, 2520, South Africa

*Corresponding author

Rasheed Adeleke, PhD

Email: rasheed.adeleke@nwu.ac.za

Phone no: +27 018 299 2495

Address: Unit for Environmental Sciences and Management, North-West University.

**ORCID**: AR, 0000-0001-6437-6419; OTE, 0000-0003-1819-8804; RA, 0000-0002-8974-422X.

# **Supplementary Tables**

**Supplementary Table 1**: Permutational multivariate analysis of variance (PERMANOVA) based on Bray-Curtis dissimilarities using the bacterial community abundance data.

|  | PERMANOVA | | | PERMDISP |
| --- | --- | --- | --- | --- |
| Fixed factors | Df | R^2^ (%) | P | P |
| Farms | 2 | 27.27 | 0.001 | 0.122 |
| Fertiliser type | 1 | 11.74 | 0.003 | 0.537 |
| Plant species | 3 | 12.75 | 0.227 | 0.964 |
| Fertiliser type: Plant species | 3 | 15.82 | 0.055 | 0.588 |

Df- degree of freedom, P < 0.05, P-value is based on 999 permutations using the adonis () function of the vegan package in R. ND; not determined.

**Supplementary Table 2**: Differentially abundant features between the farms

| Genus | LDA | P unadjusted | P adjusted | Significance |
| --- | --- | --- | --- | --- |
| *Rubrobacterales* | Farm_B | 4.350949 | 0.001959 | 0.03330 |
| *Rubrobacter* |  | 4.350949 | 0.001959 | 0.03330 |
| *Elsterales* |  | 3.568718 | 0.002101 | 0.03330 |
| *Propionibacteriaceae* |  | 3.392654 | 0.002702 | 0.03330 |
| *Virgisporangium* |  | 2.909674 | 0.002548 | 0.03330 |
| *Entotheonellaceae* |  | 2.845636 | 0.002864 | 0.03330 |
| *Segetibacter* |  | 2.412468 | 0.002835 | 0.03330 |
| *Nordella* |  | 2.348284 | 0.002835 | 0.03330 |
| *Acidobacteria* |  | 2.180584 | 0.002864 | 0.03330 |
| *Frankiales* | Farm_S | 3.879269 | 0.001959 | 0.03330 |
| *Alicyclobacillaceae* |  | 3.327737 | 0.002521 | 0.03330 |
| *Tumebacillus* |  | 3.327737 | 0.002521 | 0.03330 |
| *Rubrobacter* |  | 3.257831 | 0.001886 | 0.03330 |
| *Geminicoccaceae* |  | 3.100697 | 0.001157 | 0.03330 |
| *Bacillus* |  | 2.782937 | 0.002864 | 0.03330 |
| *Terribacillus* |  | 2.759578 | 0.002422 | 0.03330 |
| *Bdellovibrio* |  | 2.218778 | 0.002864 | 0.03330 |
| *Microtrichales* | Farm_J | 4.181742 | 0.002103 | 0.03330 |
| *Thermoactinomyces* |  | 3.571520 | 0.001649 | 0.03330 |
| *Marmoricola* |  | 3.342096 | 0.002751 | 0.03330 |
| *CL500-29 marine group* |  | 2.999481 | 0.002284 | 0.03330 |
| *Subgroup 6* |  | 2.915509 | 0.001697 | 0.03330 |
| *Kouleothrix* | Farm_T | 2.590037 | 0.000346 | 0.02171 |
| Subgroup 10 |  | 2.552849 | 0.000342 | 0.02171 |
| *metagenome* |  | 2.493913 | 0.000342 | 0.02171 |
| *Rhodopirellula* |  | 2.475376 | 0.000342 | 0.02171 |
| *Terrimonas* |  | 2.976678 | 0.002259 | 0.03330 |
| *Pontibacter* |  | 2.914398 | 0.001839 | 0.03330 |
| *UTCFX1* |  | 2.906586 | 0.000849 | 0.03330 |
| *Nocardia* |  | 2.905558 | 0.002605 | 0.03330 |
| *Bauldia* |  | 2.869715 | 0.002864 | 0.03330 |
| *Blastocatella* |  | 2.811599 | 0.000849 | 0.03330 |
| *Pseudogracilibacillus* |  | 2.751798 | 0.002057 | 0.03330 |
| *Pedomicrobium* |  | 2.731728 | 0.002057 | 0.03330 |
| *Pir4 lineage* |  | 2.728715 | 0.001071 | 0.03330 |
| *Cerasibacillus* |  | 2.709531 | 0.002864 | 0.03330 |
| *Kouleothrix* |  | 2.695504 | 0.001439 | 0.03330 |
| *Pir4 lineage* |  | 2.683881 | 0.002864 | 0.03330 |
| *Chthoniobacter* |  | 2.648163 | 0.000842 | 0.03330 |
| *Propionibacterium* |  | 2.440266 | 0.002864 | 0.03330 |
| *Herpetosiphon* |  | 2.302259 | 0.002864 | 0.03330 |
| *Oceanobacillus* |  | 2.111164 | 0.002383 | 0.03330 |

Some of the significant features (significance p ≤ 0.0333) from the 205 significant features

**Supplementary Table 3**: Differentially abundant features between organic and conventional farms

| Group (genus taxa) | LDA | P unadjusted | P adjusted | Significance |
| --- | --- | --- | --- | --- |
| *Rubrobacteria* | Organic | 4.218127 | 0.000212183 | 0.046020136 |
| Rubrobacterales |  | 4.218127 | 0.000212183 | 0.046020136 |
| *Rubrobacteriaceae* |  | 4.218127 | 0.000212183 | 0.046020136 |
| *Rubrobacter* |  | 4.218127 | 0.000212183 | 0.046020136 |
| *Elsterales* |  | 3.316274 | 0.000381058 | 0.046020136 |
| *Propionibacteriaceae* |  | 3.277556 | 0.000285118 | 0.046020136 |
| *Virgisporangium* |  | 2.955924 | 0.000212183 | 0.046020136 |
| *Geminicoccaceae* |  | 2.901468 | 0.000509639 | 0.049014667 |
| *Micrococcales* | Conventional | 4.329750 | 0.000381058 | 0.046020136 |
| *Marmoricola* |  | 3.118914 | 0.000526387 | 0.049014667 |
| CL500-29 marine group |  | 2.850692 | 0.000282117 | 0.046020136 |
| *Hymenobacteraceae* |  | 2.833395 | 0.000201679 | 0.046020136 |
| CL500-29 marine group |  | 2.746453 | 0.000504076 | 0.049014667 |
| *Luteitalea* |  | 2.712036 | 0.000341952 | 0.046020136 |
| uncultured actinobacterium |  | 2.704708 | 0.000327429 | 0.046020136 |
| *Nocardia* |  | 2.701467 | 0.000780488 | 0.049014667 |
| uncultured Chloroflexi bacterium |  | 2.549217 | 0.00074968 | 0.049014667 |
| uncultivated soil bacterium C031 |  | 2.447038 | 0.000639038 | 0.049014667 |

**Supplementary Table** **4**: Liner discriminant effect size analysis of abundant and unique pathways across the organic and conventional farms.

| Pathway comparison | LDA | P.unadj | P.adj | Sign. |
| --- | --- | --- | --- | --- |
| Metabolism\|Energy metabolism\|Sulfur metabolism | Conventional farm | 2.04953 | 0.00250 | 0.0474 |
| Metabolism\|Metabolism of other amino acids\|Phosphonate and phosphinate metabolism |  | 1.69128 | 0.00115 | 0.0311 |
| Human Diseases\|Cancers: Overview |  | 1.48840 | 0.00194 | 0.0419 |
| Human Diseases\|Endocrine and metabolic diseases |  | 1.11586 | 0.00088 | 0.0311 |
| Cellular Processes\|Transport and catabolism\|Endocytosis |  | 0.39965 | 0.00016 | 0.0170 |
| Organismal Systems\|Endocrine system\|GnRH signalling pathway |  | 0.39393 | 0.00016 | 0.0170 |
| Organismal Systems\|Immune system\|Fc gamma R-mediated phagocytosis |  | 0.37266 | 0.00016 | 0.0170 |
| Organismal Systems\|Digestive system\|Fat digestion and absorption |  | 0.23430 | 0.00115 | 0.0311 |
| Metabolism\|Amino acid metabolism | Organic farm | 3.13844 | 0.00088 | 0.0311 |
| Metabolism\|Amino acid metabolism\|Arginine and proline metabolism |  | 2.67648 | 0.00088 | 0.0311 |
| Metabolism\|Amino acid metabolism\|Phenylalanine metabolism |  | 2.40703 | 0.00115 | 0.0311 |
| Metabolism\|Energy metabolism\|Carbon fixation pathways in prokaryotes |  | 2.34942 | 0.00115 | 0.0311 |
| Metabolism\|Carbohydrate metabolism\|Citrate cycle (TCA cycle) |  | 1.84869 | 0.00194 | 0.0420 |
| Metabolism\|Metabolism of other amino acids\|Selenocompound metabolism |  | 1.82821 | 0.00115 | 0.0311 |
| Metabolism\|Xenobiotics biodegradation and metabolism\|Styrene degradation |  | 1.73386 | 0.00150 | 0.0374 |
| Human Diseases\|Infectious diseases: Parasitic\|Amoebiasis |  | 1.44485 | 0.00250 | 0.0476 |
| Metabolism\|Glycan biosynthesis and metabolism\|Glycosaminoglycan degradation |  | 1.23946 | 0.00029 | 0.0231 |

Conv; conventional farm, organic; organic farm, Sign.; significance (P < 0.05), Linear discriminant analysis; LDA P.adj; P value adjusted, P.unadj; P value unadjusted. The pathway taxonomic ranking was at level 3.

**Supplementary Table** **5**: Differentially abundant functional profile of rhizosphere bacterial community across the farms

| Functional profile group | LDA | P.unadj | P.adj | Sig. |
| --- | --- | --- | --- | --- |
| Metabolism\|Amino acid metabolism\|Phenylalanine, tyrosine and tryptophan biosynthesis | Farm_T | 2.3707 | 0.0018 | 0.0271 |
| Genetic Information Processing\|Replication and repair\|Mismatch repair | Farm_T | 2.3074 | 0.0021 | 0.0271 |
| Genetic Information Processing\|Replication and repair\|DNA replication | Farm_T | 2.0163 | 0.0025 | 0.0271 |
| Metabolism\|Energy metabolism\|Photosynthesis | Farm_T | 1.7818 | 0.0024 | 0.0271 |
| Metabolism\|Biosynthesis of other secondary metabolites\|Novobiocin biosynthesis | Farm_T | 1.3965 | 0.0020 | 0.0271 |
| Cellular Processes\|Transport and catabolism\|Endocytosis | Farm_T | 0.5468 | 0.0011 | 0.0271 |
| Organismal Systems\|Endocrine system\|GnRH signaling pathway | Farm_T | 0.5419 | 0.0011 | 0.0271 |
| Organismal Systems\|Immune system\|Fc gamma R-mediated phagocytosis | Farm_T | 0.5410 | 0.0011 | 0.0271 |
| Genetic Information Processing | Farm_T | 3.3329 | 0.0038 | 0.0277 |
| Environmental Information Processing\|Membrane transport\|Bacterial secretion system | Farm_T | 3.0245 | 0.0036 | 0.0277 |
| Genetic Information Processing\|Translation | Farm_T | 3.0002 | 0.0034 | 0.0277 |
| Metabolism\|Nucleotide metabolism | Farm_T | 2.8262 | 0.0036 | 0.0277 |
| Genetic Information Processing\|Folding, sorting and degradation\|RNA degradation | Farm_T | 2.5553 | 0.0034 | 0.0277 |
| Genetic Information Processing\|Transcription\|RNA polymerase | Farm_T | 1.7499 | 0.0043 | 0.0277 |
| Metabolism\|Glycan biosynthesis and metabolism\|Lipopolysaccharide biosynthesis | Farm_T | 2.7663 | 0.0024 | 0.0271 |
| Genetic Information Processing\|Translation\|AminOACYl-tRNA biosynthesis | Farm_T | 2.7050 | 0.0027 | 0.0271 |
| Metabolism\|Xenobiotics biodegradation and metabolism | Farm_J | 3.4191 | 0.0017 | 0.0271 |
| Metabolism\|Carbohydrate metabolism\|Butanoate metabolism | Farm_J | 2.5531 | 0.0040 | 0.0277 |
| Metabolism\|Biosynthesis of other secondary metabolites\|Stilbenoid, diarylheptanoid and gingerol biosynthesis | Farm_J | 1.8368 | 0.0042 | 0.0277 |
| Metabolism\|Metabolism of terpenoids and polyketides\|Geraniol degradation | Farm_J | 2.6789 | 0.0015 | 0.0271 |
| Metabolism\|Xenobiotics biodegradation and metabolism\|Benzoate degradation | Farm_J | 2.6467 | 0.0019 | 0.0271 |
| Metabolism\|Xenobiotics biodegradation and metabolism\|Steroid degradation | Farm_J | 2.5530 | 0.0024 | 0.0271 |
| Metabolism\|Lipid metabolism\|Fatty acid degradation | Farm_J | 2.5123 | 0.0023 | 0.0271 |
| Metabolism\|Xenobiotics biodegradation and metabolism\|Caprolactam degradation | Farm_J | 2.3068 | 0.0022 | 0.0271 |
| Metabolism\|Lipid metabolism\|Steroid hormone biosynthesis | Farm_J | 2.1687 | 0.0015 | 0.0271 |
| Metabolism\|Xenobiotics biodegradation and metabolism\|Naphthalene degradation | Farm_J | 2.1331 | 0.0019 | 0.0271 |
| Cellular Processes\|Cell growth and death\|Me–osis - yeast | Farm_J | 2.1200 | 0.0020 | 0.0271 |
| Organismal Systems\|Endocrine system\|PPAR signaling pathway | Farm_J | 2.1195 | 0.0022 | 0.0271 |
| Metabolism\|Metabolism of other amino acids\|beta-Alanine metabolism | Farm_J | 2.0362 | 0.0017 | 0.0271 |
| Metabolism\|Xenobiotics biodegradation and metabolism\|Chloroalkane and chloroalkene degradation | Farm_J | 2.0059 | 0.0024 | 0.0271 |
| Metabolism\|Lipid metabolism\|Sphingolipid metabolism | Farm_J | 1.9786 | 0.0021 | 0.0271 |
| Metabolism\|Xenobiotics biodegradation and metabolism\|Xylene degradation | Farm_J | 1.9268 | 0.0017 | 0.0271 |
| Organismal Systems\|Endocrine system\|Adipocytokine signaling pathway | Farm_J | 1.8407 | 0.0027 | 0.0271 |
| Metabolism\|Xenobiotics biodegradation and metabolism\|Dioxin degradation | Farm_J | 1.8331 | 0.0024 | 0.0271 |
| Metabolism\|Metabolism of cofactors and vitamins\|Retinol metabolism | Farm_J | 1.6107 | 0.0025 | 0.0271 |
| Metabolism\|Lipid metabolism\|Steroid biosynthesis | Farm_J | 1.4713 | 0.0026 | 0.0271 |
| Metabolism\|Lipid metabolism\|Primary bile acid biosynthesis | Farm_J | 1.3221 | 0.0017 | 0.0271 |
| Metabolism\|Biosynthesis of other secondary metabolites\|Flavone and flavonol biosynthesis | Farm_J | 0.3949 | 0.0020 | 0.0271 |
| Genetic Information Processing\|Replication and repair\|Non-homologous end-joining | Farm_J | 1.8527 | 0.0029 | 0.0275 |
| Metabolism\|Xenobiotics biodegradation and metabolism\|Polycyclic aromatic hydrocarbon degradation | Farm_J | 2.3340 | 0.0038 | 0.0277 |
| Organismal Systems\|Endocrine system | Farm_J | 2.2982 | 0.0038 | 0.0277 |
| Metabolism\|Lipid metabolism\|Synthesis and degradation of ketone bodies | Farm_J | 1.7649 | 0.0031 | 0.0277 |
| Organismal Systems\|Digestive system\|Bile secretion | Farm_J | 1.4792 | 0.0038 | 0.0277 |
| Metabolism\|Metabolism of cofactors and vitamins\|One carbon pool by folate | Farm_B | 2.0535 | 0.0030 | 0.0277 |
| Metabolism\|Glycan biosynthesis and metabolism | Farm_B | 2.8341 | 0.0028 | 0.0271 |
| Metabolism\|Metabolism of cofactors and vitamins\|Vitamin B6 metabolism | Farm_B | 1.6735 | 0.0019 | 0.0271 |
| Metabolism\|Glycan biosynthesis and metabolism\|Glycosaminoglycan degradation | Farm_B | 1.3610 | 0.0018 | 0.0271 |
| Metabolism\|Amino acid metabolism\|Valine, leucine and isoleucine degradation | Farm_S | 2.7572 | 0.0043 | 0.0277 |
| Metabolism\|Metabolism of other amino acids\|Selenocompound metabolism | Farm_S | 2.0014 | 0.0036 | 0.0277 |
| Metabolism\|Metabolism of terpenoids and polyketides\|Biosynthesis of type II polyketide products | Farm_S | 1.7453 | 0.0042 | 0.0277 |
| Metabolism\|Energy metabolism\|Methane metabolism | Farm_S | 2.7154 | 0.0061 | 0.0281 |

Linear discriminant analysis; LDA, P value unadjusted; P.unadj, P value adjusted; P.adj, Significant P value; sig.,

**Supplementary Table** **6**: Comparison of the relative abundance of selected enzymes of agroecological importance across vegetable species

| KO Terms | Enzyme and commission number | Cabbage | Lettuce | Onion | Spinach |
| --- | --- | --- | --- | --- | --- |
| K01505 | ACC deaminase [EC:3.5.99.7] | 5.13121 | 5.04508 | 4.99341 | 5.079096 |
| K02585 | nitrogen fixation protein NifB | 1.55937 | 1.56163 | 1.42423 | 1.335757 |
| K00368 | nitrite reductase [EC:1.7.2.1] | 5.28574 | 5.37568 | 5.19877 | 5.373010 |
| K01078 | acid phosphatase [EC:3.1.3.2] | 6.65048 | 6.84074 | 6.84352 | 6.953965 |
| K01077 | alkaline phosphatase [EC:3.1.3.1] | 4.87082 | 5.13687 | 5.40619 | 5.317880 |
| K01093 | 4-phytase [EC:3.1.3.26] | 0.40239 | 0.43370 | 0.39744 | 0.434442 |
| K01130 | arylsulfatase [EC:3.1.6.1] | 0.00667 | 0.00757 | 0.00768 | 0.007705 |
| K01133 | choline-sulfatase [EC:3.1.6.6] | 0.00456 | 0.00539 | 0.00538 | 0.005476 |
| K16090 | catecholate siderophore rec | 2.22708 | 2.14491 | 2.47484 | 2.213386 |
| K02217 | ferritin [EC:1.16.3.1] | 7.67035 | 7.42860 | 7.43135 | 7.249544 |
| K02013 | ICTS ATP-binding protein [EC:3.6.3.34] | 41.2667 | 42.0071 | 41.6423 | 41.99524 |
| K01183 | chitinase [EC:3.2.1.14] | 1.62722 | 1.87736 | 1.82461 | 1.819459 |
| K07405 | alpha-amylase [EC:3.2.1.1] | 5.28059 | 5.12611 | 4.62818 | 4.895040 |
| K07406 | alpha-galactosidase [EC:3.2.1.22] | 0.20130 | 0.23736 | 0.20505 | 0.210376 |
| K01179 | endoglucanase [EC:3.2.1.4] | 9.04409 | 8.67790 | 9.57702 | 8.969161 |
| K01187 | alpha-glucosidase [EC:3.2.1.20 | 5.27219 | 5.00210 | 4.96465 | 5.201447 |
| K03332 | beta-fructosidase [EC:3.2.1.80] | 3.49920 | 3.09207 | 2.97534 | 2.939153 |

The relative abundance was calculated for each enzyme across the vegetable rhizosphere soil investigated. ACC; 1-aminocyclopropane-1-carboxylate deaminase, ICTS; iron complex transport system

**Supplementary Table** **7:** Correlation between soil enzymes and physicochemical properties

| Parameter | Dehyd | Beta gluc | Alk.phos | acid phos | Urease |
| --- | --- | --- | --- | --- | --- |
| TOC | 0.413 | 0.442 | 0.028* | 0.034* | 0.771 |
| pH | 0.005* | 0.001* | 0.966 | 0.042* | 0.790 |
| Moisture | 0.283 | 0.060 | 0.823 | 0.360 | 0.139 |
| OM | 0.056 | 0.015* | 0.236 | 0.438 | 0.456 |
| EC | 0.203 | 0.344 | 0.091 | 0.023* | 0.433 |
| CEC | 0.874 | 0.378 | 0.275 | 0.435 | 0.004* |
| NO_3_ | 0.760 | 0.776 | 0.007* | 0.306 | 0.843 |
| PO_4_ | 0.018* | 0.045 | 0.336 | 0.003* | 0.626 |
| NH_4_ | 0.026* | 0.005* | 0.217 | 0.000* | 0.708 |
| SO_4_ | 0.269 | 0.939 | 0.921 | 0.476 | 0.003* |
| P | 0.792 | 0.475 | 0.885 | 0.947 | 0.003* |
| K | 0.029* | 0.019* | 0.214 | 0.000* | 0.960 |
| Na | 0.140 | 0.170 | 0.160 | 0.017* | 0.748 |
| Ca | 0.360 | 0.569 | 0.071 | 0.060 | 0.365 |
| Mg | 0.771 | 0.380 | 0.183 | 0.714 | 0.135 |
| Cl | 0.194 | 0.175 | 0.049* | 0.005* | 0.923 |
| Particle size (mm) | 0.879 | 0.409 | 0.108 | 0.304 | 0.018* |
| Clay | 0.527 | 0.098 | 0.905 | 0.491 | 0.020* |
| Sand | 0.625 | 0.251 | 0.295 | 0.972 | 0.108 |
| Silt | 0.056 | 0.418 | 0.817 | 0.278 | 0.032* |

NH_4_; ammonia, CEC; cation exchange capacity, EC; electrical conductivity, K; potassium, NO_3_; nitrate, OM; organic matter, P; phosphorus, PO4; phosphate, SO_4_; sulphate, and TOC; total organic carbon, “*” Significant (P < 0.05) value. Dehydogenase; Dehyd., Alkaline phosphatase; Alk.phos, acid phosphatase; acid.phos, beta glucosidase; Beta.gluc.

**Supplementary Table 8**: Significant environmental variables correlating with the bacterial communities (genus taxa) within the redundancy analysis triplot.

| Variables | Variance | F | Pr(>F) | Mantel test | | |
| --- | --- | --- | --- | --- | --- | --- |
|  |  |  |  | P value | adj. P | Sig. |
| TOC (total organic carbon) | 2648870 | 3.657678 | 0.001 | 0.2901 | 0.009 | 0.0210 |
| pH | 671366.8 | 0.927053 | 0.495 | -0.106 | 0.938 | 0.9380 |
| Moisture content | 873237.3 | 1.205805 | 0.280 | -0.020 | 0.528 | 0.5686 |
| OM (organic matter) | 3980192 | 5.496026 | 0.001 | 0.1665 | 0.041 | 0.0718 |
| EC (electrical conductivity) | 2023697 | 2.794411 | 0.015 | 0.3151 | 0.006 | 0.0210 |
| CEC (cation exchange capacity) | 1945933 | 2.687031 | 0.020 | 0.0786 | 0.114 | 0.1330 |
| NH4 (ammonia ion) | 444958.9 | 0.614419 | 0.780 | 0.1240 | 0.078 | 0.1092 |
| SO4 (sulphate ion) | 599286.3 | 0.827521 | 0.574 | 0.3118 | 0.008 | 0.0210 |
| PO4 (phosphate ion) | 313507.4 | 0.432905 | 0.925 | 0.1177 | 0.092 | 0.1171 |
| Na (sodium) | 655668.9 | 0.905377 | 0.511 | 0.1933 | 0.030 | 0.0600 |
| Mg (magnesium) | 926024 | 1.278695 | 0.268 | 0.3023 | 0.004 | 0.0210 |
| Clay | 876681.4 | 1.210561 | 0.303 | 0.2721 | 0.005 | 0.0210 |
| Sand | 1124022 | 1.552099 | 0.149 | 0.3145 | 0.008 | 0.0210 |
| Silt | 296185.7 | 0.408986 | 0.936 | 0.1214 | 0.073 | 0.1092 |

|  |  |  |  |  |
| --- | --- | --- | --- | --- |

Permutation test for RDA under the reduced model. The terms are added sequentially (first to last) and the number of permutations is 999. Degree of freedom = 1. The R^2^ = 0.8275777 and adjusted R^2^ = 0.3447952. Mantel check was used to check for significant correlations between environmental variables and distance matrix, adj. P; adjusted P values, Sig; significant value.

## **Supplementary Figures**

**Supplementary Figure 1**: Rarefaction curve for rhizosphere bacterial communities from different farms. Subsampling of the community was done at an even depth of 74170 sequences per sample. The plateau in the sequence graph indicates that the richness of the community was adequately represented.

**Supplementary Figure 2**: Comparison of shared and unique amplicon sequence variants (ASVs) in (A) organic farms and (B) conventional farms

**Supplementary Figure 3**: Unique and shared amplicon sequence variants (ASVs) across the rhizosphere soil of vegetable crops

**Supplementary Figure 4**: Bray-Curtis dissimilarity of bacterial communities of rhizosphere soil from different plant species based on non-metric multidimensional scaling (NMDS). The eclipses in the NMDS plot show 95% confidence intervals (standard error) in multivariate space within the group centroids, The stress plot (Supplementary Figure 5B) for the NMDS showed that the original dissimilarities are well preserved (stress = 0.165047) in the reduced number of dimensions

**Supplementary Figure 5**: Rhizosphere bacterial community structure variability in conventional and organic farms (A) Average distance of the community dispersion to the median and (B) stress plot. The higher median indicates a high probability of higher community dispersion in organic farms compared to conventional farms. The stress p000000000lot was generated from the non-metric multidimensional scaling plot and was constructed with the stress plot () function of the vegan package in R software. The stress was completed at 0.1650471.

**Supplementary Figure 6**: Average relative abundance of the dominant bacterial community in the rhizosphere soil of different vegetables (A) Dominant phyla (B) Dominant genera taxa level. The phylotypes with average relative abundance below 1% and the unculturable and unclassified at the genus taxa level constitute the others. The bar plots were constructed based on average relative abundance per plant type.

**Supplementary Figure 7**: Correlation between species function percentage of bacterial community with soil properties. NH_4_; ammonia, TOC; total organic carbon, EC; electrical conductivity, CEC; cation exchange capacity, OM; organic matter
