# Supplementary figures and images for: Soil nutrient management influences diversity, community association and functional structure of rhizosphere bacteriome under vegetable crop production
Source: Front Microbiol. 2023 Sep 28;14:1229873. doi: 10.3389/fmicb.2023.1229873 (PMC10568080; doi:10.3389/fmicb.2023.1229873)

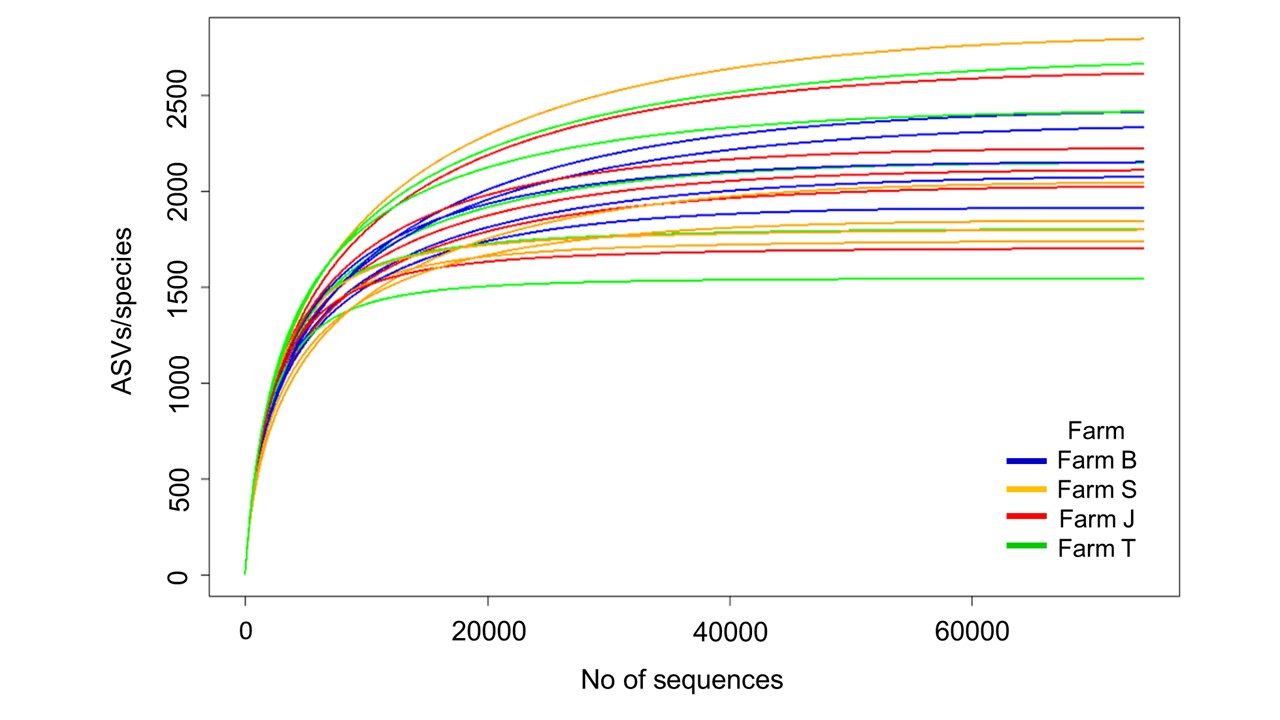

Supplement: Supplementary file 2 [file Image_1.JPEG]

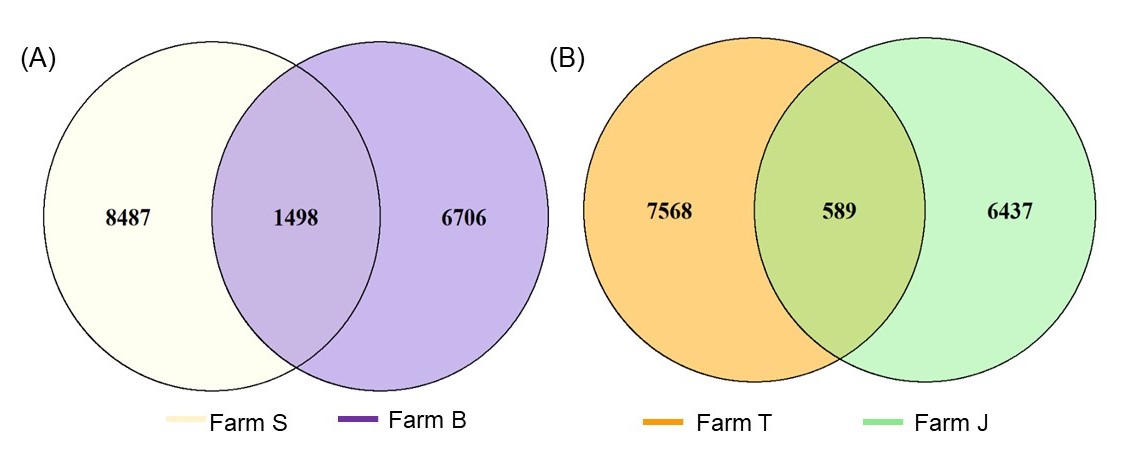

Supplement: Supplementary file 3 [file Image_2.JPEG]

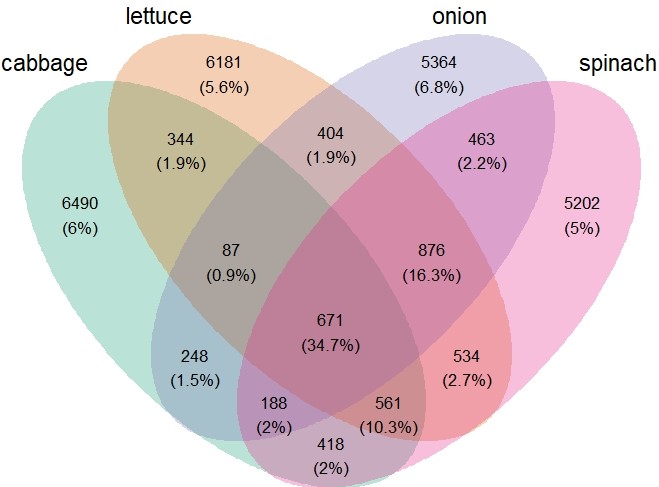

Supplement: Supplementary file 4 [file Image_3.JPEG]

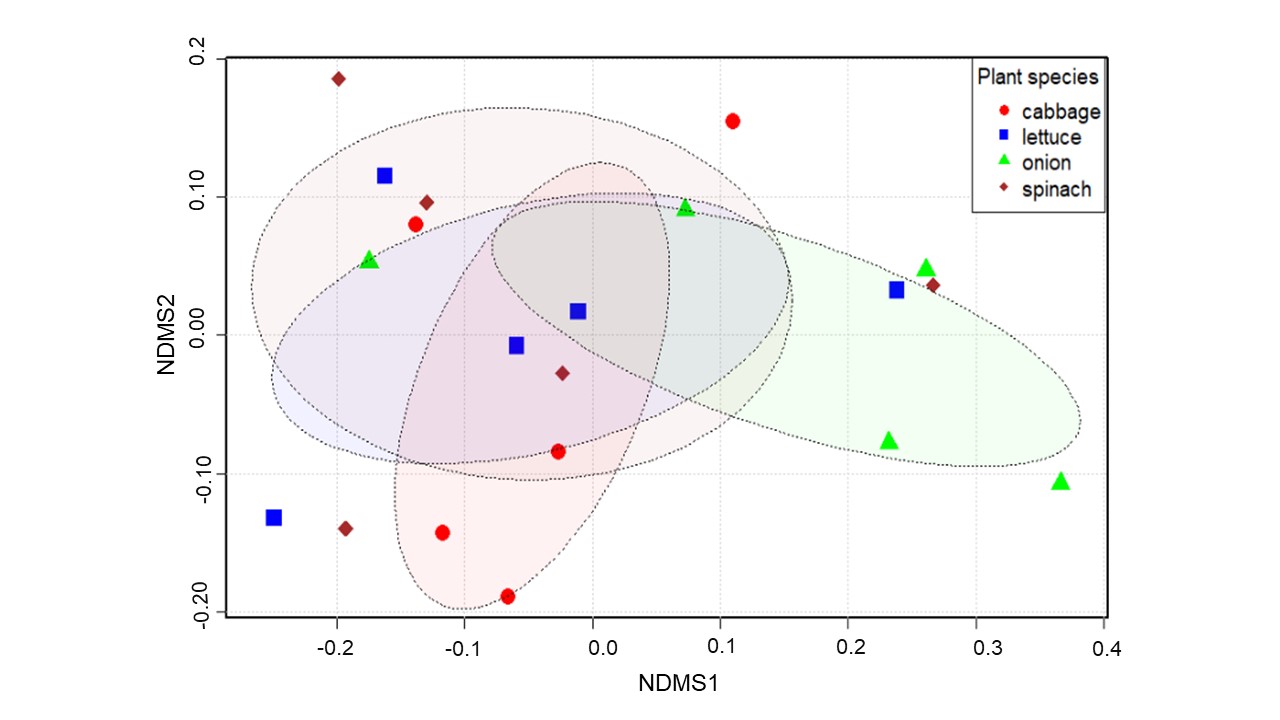

Supplement: Supplementary file 5 [file Image_4.JPEG]

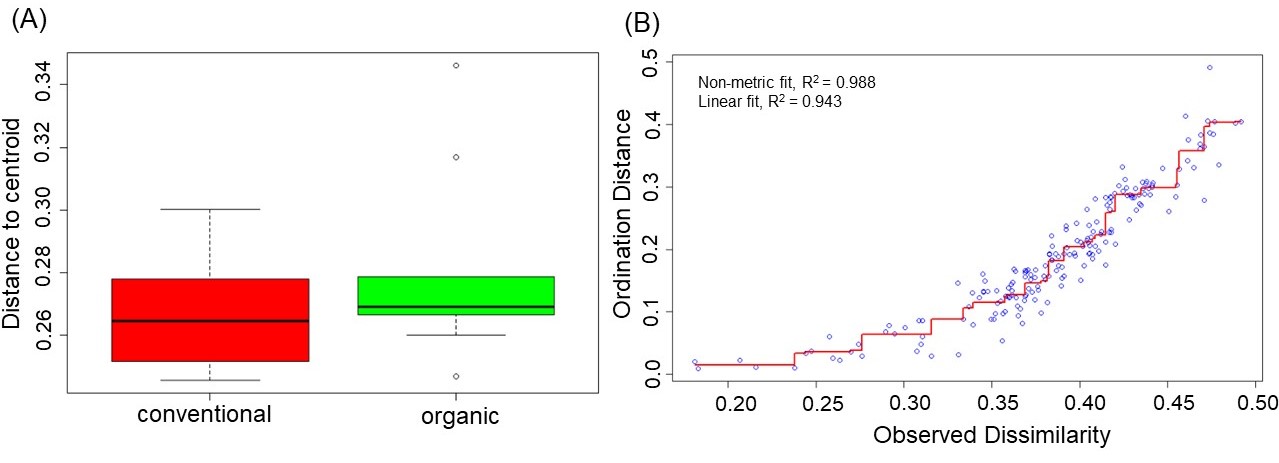

Supplement: Supplementary file 6 [file Image_5.JPEG]

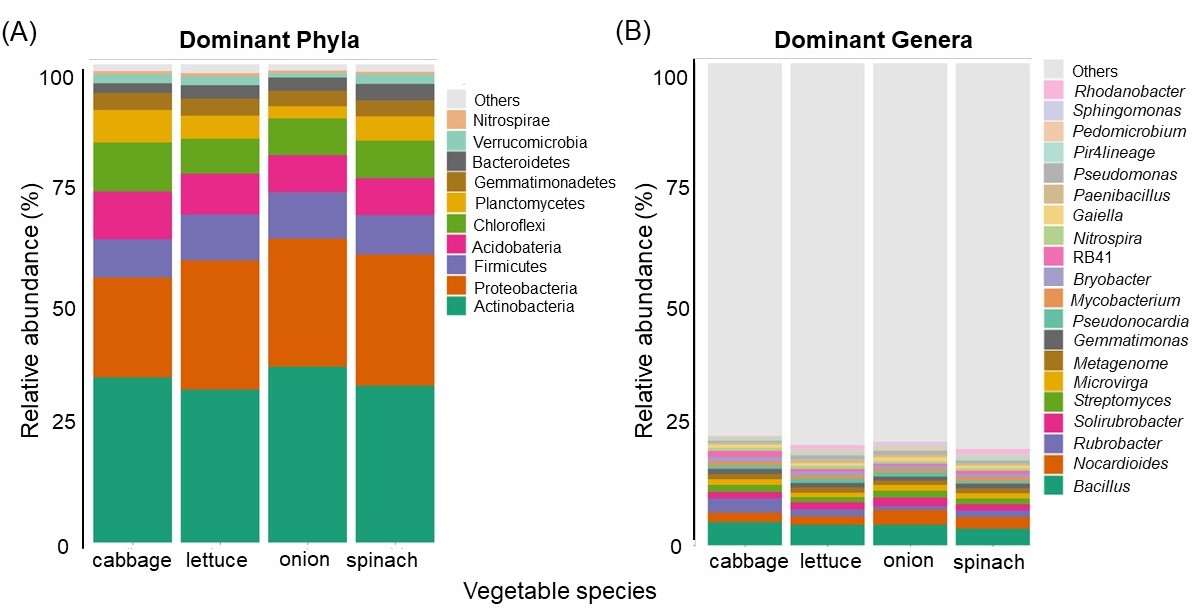

Supplement: Supplementary file 7 [file Image_6.JPEG]

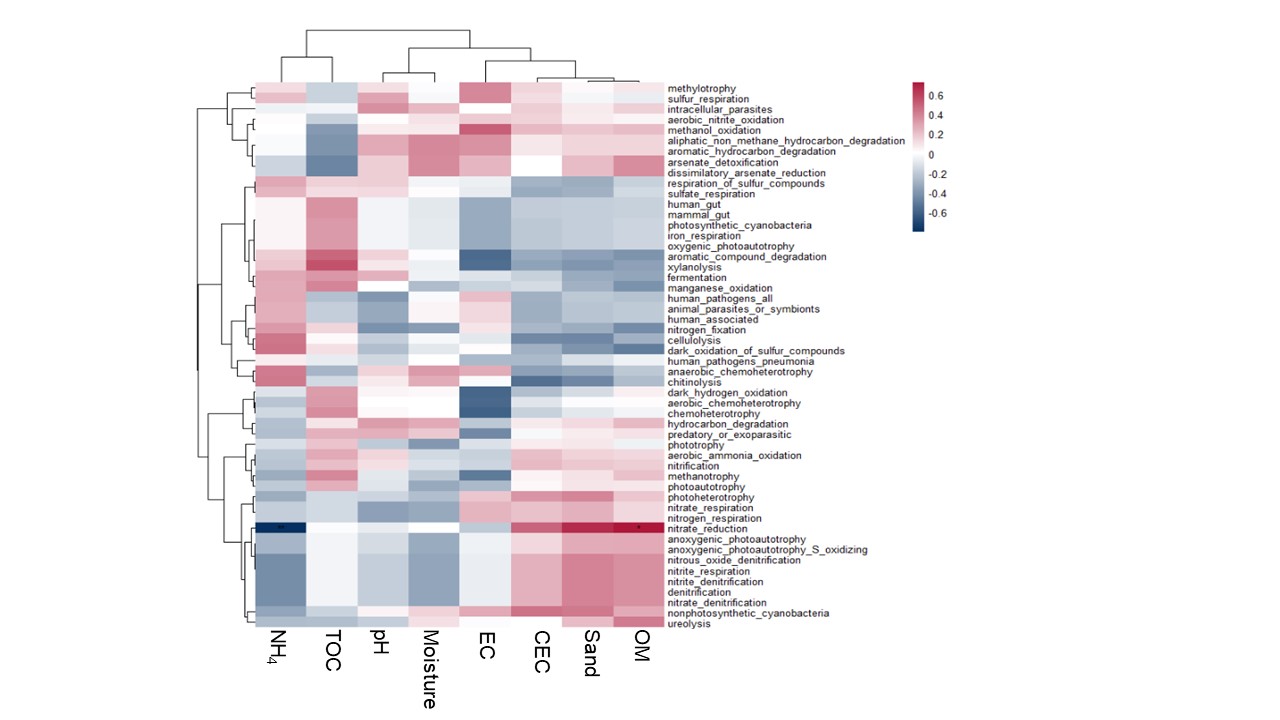

Supplement: Supplementary file 8 [file Image_7.JPEG]
